# Is UK Puppy Purchasing Suffering a Long COVID Effect? Ongoing Negative Impacts of the COVID-19 Pandemic upon Puppy Purchase Motivations and Behaviours in 2021

**DOI:** 10.3390/ani13132186

**Published:** 2023-07-03

**Authors:** Rowena M. A. Packer, Claire L. Brand, Zoe Belshaw, Camilla L. Pegram, Fiona Dale, Kim B. Stevens, Dan G. O’Neill

**Affiliations:** 1 Department of Clinical Science and Services, The Royal Veterinary College, Hatfield AL9 7TA, UK; clbrand@rvc.ac.uk; 2 EviVet Evidence-Based Veterinary Consultancy, Nottingham NG2 5HY, UK; z.belshaw.97@cantab.net; 3 Department of Pathobiology and Population Sciences, The Royal Veterinary College, Hatfield AL9 7TA, UK; cpegram@rvc.ac.uk (C.L.P.); fidale@rvc.ac.uk (F.D.); kstevens@rvc.ac.uk (K.B.S.); doneill@rvc.ac.uk (D.G.O.)

**Keywords:** dogs, puppy, COVID-19, lockdown, welfare, human–animal interaction, pandemic puppies, dog ownership, dog breeding

## Abstract

**Simple Summary:**

The COVID-19 pandemic resulted in widescale changes to how UK puppy buyers purchased their puppies. Many of these changes threatened canine welfare by increasing the likelihood of buyers purchasing a puppy from a low-welfare source, e.g., collecting a puppy away from its place of birth or without seeing their mother, both of which are currently illegal in the UK. However, whether these puppy-purchasing changes were limited only to the 2020 ‘peak pandemic’ phase and had returned to their pre-pandemic baseline afterwards or had persisted into the later phases of the pandemic in 2021 was not known. This study explored how and why puppies were purchased in 2021 and then compared this to the same date-periods in 2020 and 2019. Valid responses were analysed from the owners of 1148 “2019 puppies”, 4369 “2020 Pandemic Puppies”, and 2080 “2021 puppies”. Although some of the peak pandemic changes had returned to pre-pandemic levels in 2021, some worrying trends still persisted, including 2021 puppies being less likely to be viewed in-person pre-purchase or to be collected from inside their breeders’ property, compared to 2019 puppies. Furthermore, concerning year-on-year increases were documented between 2019 and 2021 in the number of puppies being sold with a passport indicating importation to the UK, often under the minimum legal age for import. The issues identified in this paper require further monitoring and intervention to protect canine welfare in the future.

**Abstract:**

The COVID-19 pandemic led to a surge in acquisitions of puppies in the UK, dubbed the “Pandemic Puppy” phenomenon. In addition to an increased demand for puppies, widespread changes to both why and how puppies were purchased during this period compared to pre-pandemic 2019 purchases were documented, many of which threatened canine welfare (e.g., puppies being collected away from their place of birth, without seeing their mother). This study aimed to explore which changes to the pre-purchase and purchase motivations and behaviours of UK owners who purchased a puppy aged <16 weeks in the 2020 phase of the COVID-19 pandemic had persisted into 2021 or had returned to pre-pandemic 2019 levels. An online survey was conducted during February to April 2022 from which 2080 valid responses were analysed (“2021 puppies”) and compared with previously collected data from comparable cohorts in 2019 (*n* = 1148, “2019 puppies”) and 2020 (*n* = 4369, “Pandemic Puppies”). While the majority of the peak pandemic changes documented in 2020 had returned to their 2019 pre-pandemic baseline, others persisted into 2021. Multinomial logistic regression models revealed that the shifts during 2020 towards owners viewing their puppy pre-purchase over video calls or via video recordings/photos rather than in-person and towards collecting their puppy from outside of their breeders’ property rather than inside had persisted into 2021 and had not returned to pre-pandemic levels. Year-on-year significant rises in the number of puppies sold with a passport were documented between 2019 and 2021, with over 1 in 10 2021 puppies having been sold with a passport, the figure more than doubling since 2019. An increasing number of these puppies sold with a passport were under the minimum legal age for import at sale. Going forward, these concerning changes require further monitoring and human behaviour change interventions to tackle, including increased buyer awareness but also legislative approaches to prevent the greatest harm.

## 1. Introduction

The COVID-19 pandemic gave rise to an international shift in the volume of adult dogs and puppies that were acquired by domestic households, the latter dubbed the “Pandemic Puppy” phenomenon [1]. Although challenging to quantify precisely, multiple data sources point towards significant increases in the acquisition of adult dogs and/or puppies, or interest in acquiring them, particularly during the early phases of the COVID-19 pandemic (Spring 2020). Global Google Trends analysed documented international surges in the relative search volume for dog adoption in 2020 in countries including (in descending order of public interest) the USA, Australia, Singapore, Canada, New Zealand, and the UK [2]. Peak interest in dog adoption in that study was observed in April 2020, the early epidemic phase of the COVID-19 pandemic [2]. In a similar Google Trends study, this time focusing upon puppy acquisition specifically, lockdowns in all five countries investigated (Australia, Canada, Germany, UK and USA) resulted in a significant increase in online puppy searching [3]. Although not all searches for puppies or adult dogs are likely to have resulted in actual acquisitions (either via purchase or adoption), several international studies indicated that adult dog and/or puppy acquisitions increased during the COVID-19 pandemic, including in Israel [4] and Australia [5], the latter following an RSPCA Australia-led “*clear the shelter*” campaign, which was highly successful in increasing pet adoptions, combined with the documented effects of government lockdowns [6].

Correspondingly, estimates of international pet populations increased during this period. The Pet Food Manufacturers Association reported that 3.2 million households acquired a puppy during the pandemic in the UK [7], and similarly, over 2 million households were reported to have acquired a pet during the pandemic in Australia [8]. Both the USA and Canada reported increases in pet ownership, including a reported 18% increase in households owning a pet in Canada [9] and 6% increase in households owning a dog in the USA, with the American Pet Products Association reporting an increase from 48% to 54% of households owning dogs [10]. Although some of the aforementioned pandemic-pet acquisitions are likely to have been companion animal species other than dogs (e.g., cats) or were the result of adoptions from rehoming charities, some evidence points towards an increase in puppy purchases within these reported figures. The German Kennel Club (VDH) reported a 20% increase in dog purchasing during 2020 compared with previous years [11], and The Kennel Club (UK) reported a 168% increase in searches using their ‘Find a Puppy’ tool during the early phase of the pandemic (23 March and 31 May 2020) compared to the same period in 2019 [12]. This UK trend was reflected in the recent Dogs Trust ‘National Dog Survey’ of 440,423 dogs, where the boom in acquisition of younger dogs during the pandemic was evident in the large number of dogs aged under 2 years old in the survey (26% of the entire sample) [13].

In many cases, this shift towards increased purchase, adoption and fostering of dogs during periods of pandemic-induced social isolation was framed positively, for example, Parry stated: “*This is good news for people and pets who now get to enjoy the great advantages of the human–animal bond during this unique time of great need*” [14]. Indeed, many studies have since reported benefits of ownership of dogs and other companion animals (acquired prior to the pandemic) in supporting the mental health of their adult owners during the pandemic, both in the UK and worldwide [15,16,17,18,19,20]. Owner-reported benefits of dog ownership during this period included mood enhancement and stress reduction that helped owners cope with the COVID-19 lockdown phase(s), giving owners a sense of purpose, focus, motivation and routine, reducing feelings of loneliness, offering distraction and a sense of normalcy, and increasing engagement in physical activity [21]. Our own study of Pandemic Puppy owners found that during the 2020 phase of the pandemic, puppies were often purchased in attempts to improve pandemic-related mental health challenges in the household, with three of the top five reasons as to why the pandemic influenced puppy purchases being mental health related, e.g., companionship while spending increased time at home, exercise encouragement, and a new ‘happy’ focus [1].

Despite what was often reported as a positive phenomenon in the media, and a positive for human mental wellbeing, pandemic-related dog acquisitions are likely to also have had some unintended negative impacts on the welfare of the dog population, due to changes in how pets were acquired during this period. In the USA, the American Pet Products Association reported that compared to their 2017–2018 statistics, the proportion of dogs acquired from rescue organizations decreased during the pandemic, whilst the proportion acquired from pet stores and breeders increased [22]. This change may reflect the demand for rescue animals outpacing the number of animals available for adoption due to reduced relinquishments during this period, limitations to rescue center services during the pandemic, or other owner motivational factors (e.g., increased desire for puppies over adult dogs, or specific breeds unlikely to be found in rescue centers). These findings were echoed in a large UK sample collected by Dogs Trust, who found that compared with owners who acquired their dog between 2018–2019, owners who acquired their dog during the pandemic (between 2020–2021) were less likely to source their dog from a rehoming centre, either online (−2.3%) or via in-person centre visits (−3.0%), and in contrast, were more likely to source their dogs from pet websites (+7.6%) and family friends (+2.0%) [13]. This shift towards private, often online sales, of puppies led to high demand that could not be fully met by good welfare sources, resulting in increased sales from unscrupulous breeders and sellers, and illegal importers of puppies [1]. The strong demand for puppies from private sellers was reflected in inflated purchase prices; our own study found that owners of Pandemic Puppies purchased from 23 March–31 December 2020 paid significantly more for their puppy than puppies purchased during the same date-range in 2019, with one quarter of Pandemic Puppy owners (24.3%) paying £2000–2999 for their puppy compared to just 1.8% of 2019 puppy owners [1]. Similarly, Dogs Trust research also found that paying over £2001 for a puppy only became prevalent during the COVID-19 pandemic [13], and online selling platform Pets4Homes found that the average price of puppies for sale in 2020 was £1875, compared to £808 in 2019, a +131% increase [23].

Our research team conducted a large-scale investigation of puppy purchasing motivations and behaviours during the pandemic in the UK, comparing puppies purchased aged under 16 weeks from private sellers between 23 March–31 December 2020 with those purchased during the same date-range in 2019 [1,24]. Although suboptimal puppy-purchasing behaviours were evident in the UK prior to the pandemic [25], including deficits in pre-purchase research [26], purchase of puppies that were underage at time of sale [27], without seeing their mother [27], and without any pre-purchase visits [26], the COVID-19 pandemic exacerbated some of these pre-existing practices, and introduced new concerning trends in UK puppy purchasing behaviours. Key findings included Pandemic Puppy owners being less likely to view their puppy in-person, instead shifting to ‘virtual viewings’ (e.g., video calls, video recordings and photographs), likely a reflection of movement and social-distancing restrictions during the peak phases of the pandemic [1]. Furthermore, Pandemic Puppy owners were more likely to pay a deposit without physically seeing their puppy than 2019 owners, potentially a reflection of the strong competition to secure a puppy during times of peak interest [1] (e.g., Pets4Homes reported 420 buyers competing for every pet for sale in May 2020 [23]). Pandemic Puppy owners appeared less focused upon canine health, being less likely to seek out a breeder that performed health testing on their breeding dog(s), and once a breeder had been found, being less likely to ask to see any information regarding health testing of their puppy’s parents (either DNA [genetic] or veterinary screening tests [e.g., hips, elbows, knees, eyes, or respiratory] testing) [1]. At purchase, Pandemic Puppies were more likely to be younger, delivered or collected from outside their breeders’ property, and be seen without their mother or their littermates, despite Lucy’s Law being passed into English legislation in 2019, which made it illegal to sell a puppy without showing it with its mother in the place the puppy was born [28]. These sub-optimal purchasing behaviours left owners vulnerable to purchases from unscrupulous breeders and dealers, including puppies sourced by illegal importation and/or reared on puppy farms [29]. Although still under investigation in the 2020 Pandemic Puppy cohort as part of an ongoing longitudinal study [30], from existing literature, it is possible that this vulnerable population of dogs will experience long-term negative consequences upon their behaviour and welfare [31,32,33,34].

In addition to changes to puppy purchasing behaviours, shifts in the demographic profiles and experience levels of households that purchased puppies during the pandemic were also identified across several data sources. Pandemic Puppy owners were more likely to be first-time dog owners (2 in 5) compared to 2019 puppy owners (1 in 3) [1]; this relative inexperience may pose risks to the future welfare of these dogs, including their behaviour [35,36,37,38], dog–owner bond [39] and relinquishment risk [40,41]. Over 10% of Pandemic Puppy owners had not considered purchasing a puppy before the pandemic, with 2 in 5 reporting that their decision to purchase a puppy had been explicitly influenced by the pandemic, most commonly by perceptions of having more time to care for a dog (86.7%) [1]. These driving factors for acquisition may have led to vulnerabilities in households where, for example, the additional free-time experienced during the pandemic was liable to reduce once restrictions were lifted [42]. Further differences to demographics included Pandemic Puppies being more likely to be purchased by households that included children, particularly younger children aged 5–10 years [1]. This finding was echoed in data from the USA showing that households with children were 1.83 times more likely to have acquired an animal during the pandemic than those without [22]. Both this US study [22] and UK data from Dogs Trust [13] further found that younger people (aged 18–34) were more likely to acquire a dog during the pandemic than those in older age groups.

Whether the widespread changes to puppy purchasing reported during the 2020 phase of the COVID-19 pandemic were acute and temporary (e.g., restricted to the initial surge in interest in puppies in Spring 2020), circumstantial (e.g., related to lockdown restrictions, some of which persisted into 2021: Figure 1), or have fully returned to pre-pandemic levels following cessation of restrictions in mid-2021 is not yet known. However, this knowledge is crucial to protecting canine welfare, given the potential lifelong consequences of poor purchasing behaviours upon dogs, many of which increased in 2020. Identifying persistent negative changes to puppy purchasing culture would allow for bespoke campaigning and interventions on key areas of concern.

This study aimed to explore whether the changes to puppy purchasing in the UK documented during the 2020 phase of the COVID-19 pandemic had persisted into 2021 or had returned to pre-pandemic 2019 levels. Using a cross-sectional analysis of a national survey, we sought to:

(i) describe the pre-purchase motivations and behaviours and purchase behaviours of owners of puppies purchased 23 March–31 December 2021;
(ii) compare the data from (i) with the motivations and behaviours of owners of puppies purchased during the same date frame in 2019 and 2020.

## 2. Materials and Methods

### 2.1. Survey Design and Content

An online questionnaire previously developed and launched by the Pandemic Puppies research team to explore the pre-purchase and purchase motivations and behaviours of UK puppy purchasers before the COVID-19 pandemic (23 March–31 December 2019) and during the early phase of the pandemic (23 March–31 December 2020) [1] was repeated for a cohort of owners who purchased a puppy during the same date-period in 2021. Survey content was repeated from the previous study to allow for the direct comparison of results; however, several new fixed-choice responses were added to multiple-choice questions. These responses were designed based on qualitative content analysis of free-text [43] in response to the option “Other, please specify”, inductively generating new response options based on novel owner insights, as previously described [1]. The same five overarching sections were included: (1) general owner demographics; (2) general puppy demographics; (3) pre-purchase motivations; (4) pre-purchase behaviours; and (5) purchase behaviours. Owners of 2021 puppies were asked an additional set of questions regarding COVID-19 specific impacts previously posed to 2020 Pandemic Puppy owners.

To increase simplicity for respondents, references were made to the puppy’s ‘breeder’ throughout the survey (which is reflected throughout this paper); however, despite laws prohibiting this [28], it is possible that some ‘breeders’ did not actually breed the puppies they were selling, and instead may be considered puppy dealers/third party sellers, which puppy purchasers may or may not have been aware of at the time of sale.

This study received ethical approval from the Social Science Research Ethical Review Board at the Royal Veterinary College (URN: SR2022-0004).

### 2.2. Participant Recruitment

The survey was open for 7 weeks from 14 February 2022 to 6 April 2022, and was hosted on SurveyMonkey. To reduce bias between the current sample and comparative (2019–2020) samples, the same sampling frame and recruitment methods were employed across both surveys. Distribution via snowball sampling was achieved via sharing from a wide range of sources, including social media, the veterinary, canine, and general press, and through key stakeholders including the commercial and charity sectors. Respondents provided informed consent for their data to be held on a secure server in accordance with UK GDPR legislation. IP addresses were used to eliminate duplicate responses prior to being permanently deleted.

Inclusion criteria were consistent with the 2019–2020 survey [1], with respondents required to be UK residents aged 18 years or older and to have brought home a puppy aged under 16 weeks purchased at any date during 2021 (N.B. although all puppy purchases in 2021 were eligible for the broader project, responses analysed in this study were limited to owners who brought home their puppy between 23 March–31 December 2021 in order to align with analysed samples from 2019 and 2020). Owners were required to have purchased their puppy rather than having bred the puppy themselves or having rehomed it. If owners had purchased more than one puppy during 2021, they were requested to answer for the youngest puppy at the time of the survey, and if they had purchased littermates, to answer for the dog whose name came first alphabetically.

### 2.3. Data Cleaning

Raw survey data were cleaned in Microsoft Excel, which included the removal of responses that did not meet inclusion criteria, duplicate responses based on IP address, and responses with no data beyond the consent.

### 2.4. Spatial Analysis

Partial postcode data were requested from respondents in order to assess the representativeness of the 2021 sample to the UK population. Following verification of their validity against the Office for National Statistics (ONS) National Statistics Postcode Lookup (NSPL) 2021 data, partial postcodes were assigned to one of the 12 UK regions. Regional response rates were then calculated as:

$$\frac{Total\;responses\;from\;a\;region\;in\;a\;year}{ONS\;region\;population}\times 100000$$

Choropleth maps were produced using ArcGIS 10.2 (Environmental Systems Research Institute, Redlands, CA, USA) to show the regional response rates per 100,000 population for 2021, and the percentage difference in response rates between the 2021 data and both 2019 and 2020 data.

### 2.5. Qualitative Content Analysis of Free-Text Options

Many questions included an “Other, please specify” option to supplement fixed-choice responses to multiple-choice questions (MCQs). Where these responses were considered to fit within an existing fixed-choice response (which were extensive, following the addition of new categories from qualitative content analysis of 2019–2020 data [1]), these data were back-allocated to the most appropriate response, if it had not already been selected by the respondent. To allow for the inclusion of new response categories, where appropriate, any remaining free-text responses were subject to qualitative content analysis [43], as previously described [1].

### 2.6. Quantitative Analysis

Following cleaning in Excel, data were imported into IBM SPSS Statistics v27 (SPSS Inc, Chicago, IL, USA). Following the calculation of descriptive statistics (frequency and percentage) for all variables, univariable comparisons were made between the three puppy cohorts: (i) puppies purchased between 23 March–31 December 2019 (“2019 puppies”); (ii) puppies purchased between 23 March–31 December 2020 (“Pandemic Puppies”), and (iii) puppies purchased between 23 March–31 December 2021 (“2021 puppies”). Chi-squared (*X*^2^) analysis was applied for categorical variables, with Bonferroni-corrected post-hoc comparisons made between individual years, and differences highlighted in tables as subscript letters (e.g., a, b, c, if all three years differed significantly from each other). Mann–Whitney U-tests were applied for non-normally distributed continuous variables (with data distribution ascertained by visual inspection of histograms).

Variables liberally associated with acquisition year in the univariable analyses (*p* < 0.2) were included in four separate multinomial regression modelling exercises describing (i) demographics, (ii) pre-purchase motivations, (iii) pre-purchase behaviour, and (iv) purchase behaviour, with year of acquisition (2019, 2020, 2021) as the outcome. Variable responses that were newly added as categories in the 2021 survey based on content analysis of free-text responses in the 2019–2020 cohorts were not included in multivariable models due to this difference in data collection method potentially influencing these results. Models were developed using manual backwards stepwise elimination, with confounding assessed for all variables retained in the final models by adding each variable to the model in a stepwise manner and checking for substantial changes (>20%) to the odds ratio (OR) or any other independent variable following its addition [44]. Furthermore, collinearity of variables was evaluated through inspection of correlation matrices, tolerance and variance inflation factor (VIF) [45]. Interactions between all independent variables in the final models were assessed for significance. The Hosmer–Lemeshow test was used to evaluate the quality of the model fit.

Multivariable binary logistic regression models were constructed to explore the impact of acquisition year and month (and the interaction between these two variables) upon the likelihood of (i) *not* viewing a puppy in person pre-purchase, (ii) *not* collecting a puppy from inside the breeder’s property, and (iii) a puppy being sold with a passport.

Statistical significance was set at the 5% level.

## 3. Results

In total, *n* = 2827 responses were returned for the survey of 2021 puppies. Following cleaning, removals included *n* = 10 responses due to duplication, *n* = 275 responses that held no data beyond the consent and inclusion criteria, *n* = 19 responses that did not meet the inclusion criteria (of which *n* = 13 had adopted their puppy rather than purchased it, *n* = 5 who had bred the puppy themselves and *n* = 1 who purchased their puppy in 2019). This resulted in a remaining valid sample of *n* = 2523 puppies, of which *n* = 2080 were purchased between 23 March–31 December 2021 (“2021 puppies”) that are included in analyses here, compared with previously collected data [24] from comparable cohorts in 2019 (*n* = 1148, “2019 puppies”) and 2020 (*n* = 4369, “Pandemic Puppies”).

### 3.1. Spatial Analysis

**Figure 2:**
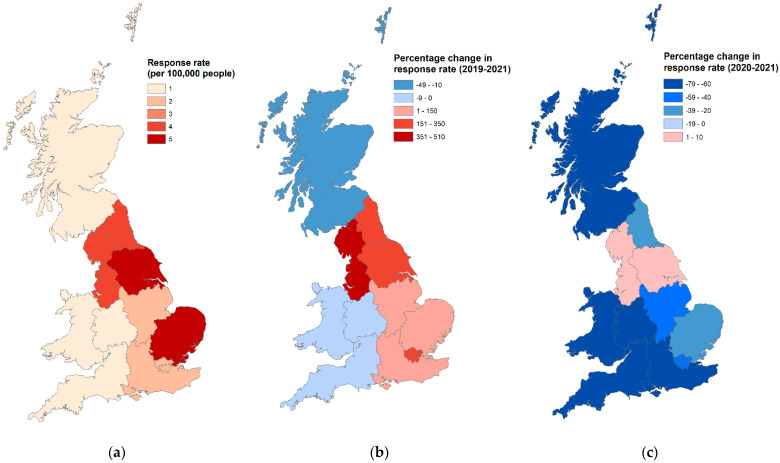
Choropleth maps of the UK describing the distribution of respondents who gave postcode data. (**a**) Respondents per 100,000 of UK population 2021 (*n* = 946); (**b**) percentage change of response rates between 2019 and 2021; (**c**) percentage change of response rates between 2020 and 2021.

The geographical distribution of respondents by year is depicted in Figure 2, including changes between years (2019 vs. 2021, 2020 vs. 2021). No significant differences in the geographical distribution of the sample compared with Office for National Statistics population data for mid-2021 were found (t = 0.001, df = 11, *p* = 0.500). Compared to pre-pandemic 2019 owners, owners of 2021 puppies were significantly less likely to live in Scotland (2021: 15.86% vs. 2019: 4.02%) but significantly more likely to live in the North West (2021: 16.08% vs. 2019: 5.39%) or Yorkshire and The Humber (2021: 14.72% vs. 2019: 7.08%; *p* < 0.001). Compared to 2020 Pandemic Puppy owners, the owners of 2021 puppies were significantly more likely to live in the North West (2021: 16.08% vs. 2020: 8.13%), Yorkshire and the Humber (2021: 14.72% vs. 2020: 7.76%) but less likely to live in the South East (2021: 9.66% vs. 2020: 16.11%) or the South West (2021: 4.54% vs. 2020: 10.95%; *p* < 0.001).

### 3.2. Owner Demographics and Lifestyle

Owners of 2021 puppies were predominantly female (94.8%), with a significantly higher proportion of female owners in 2021 compared to 2020 (90.0%) or 2019 (92.0%) (*X*^2^ = 54.96, *p* < 0.001). The most common age group of 2021 owners was 45–54 years old (24.1%). The 2021 owners were overall older than 2020 owners and were less likely to be 25–34 years old (20.5%) than 2020 owners (24.1%) but more likely to be 55 to 64 years old (19.2%) than 2020 owners (15.6%) (*X*^2^ = 30.36, *p* = 0.002), returning to pre-pandemic (2019) levels. The majority of 2021 owners were the primary carer for their puppy (i.e., providing the majority of care such as feeding and walking; 65.9%), with 2021 owners being less likely to share the caring role with someone else in their household (31.6%) than 2020 puppy owners (39.8%; *X*^2^ = 56.62, *p* < 0.001), returning back to pre-pandemic (2019) levels (34.0%, *p* > 0.05).

#### 3.2.1. Experience with Dogs

Owners of 2021 puppies were more likely to have previously owned a dog (66.7%) than 2020 Pandemic Puppy owners (59.6%; *X*^2^ = 33.63, *p* < 0.001), returning to pre-pandemic (2019) levels (66.6%, *p* > 0.05). There was no difference in proportion between owners of 2021 puppies, 2020 puppies and 2019 puppies that had grown up with a dog (2019: 68.1% vs. 2020: 69.3%; 2021: 67.9%; *X*^2^ = 0.71, *p* = 0.701). Owners of 2021 puppies were more likely to be employed in the canine and/or animal care sector (16.6%) compared with 2020 puppy owners (10.0%; *X*^2^ = 87.82, *p* < 0.001), returning to pre-pandemic (2019) levels (17.9%; *p* > 0.05).

#### 3.2.2. Household Demographics

Household demographic changes that were documented during the pandemic between 2019 to 2020 had returned to pre-pandemic 2019 levels in 2021. Puppies purchased in 2021 were less likely to live in households with both adults and children (28.2%) compared to 2020 puppies (33.2%; *X*^2^ = 22.32, *p* < 0.001), returning to pre-pandemic (2019) levels (26.8%; *p* > 0.05). Within those households with children, no differences were detected between the age profiles of children living with 2021 puppies vs. 2020 or 2019 puppies. The previously documented increase in households with 5–10-year-olds in 2020 (50.3%) returned to pre-pandemic (2019) levels in 2021 (2021: 44.9%, 2019: 37.7%; *p* > 0.05). Similarly, the previously documented decrease in households with 16–18-year-olds in 2020 (24.7%) returned to pre-pandemic (2019) levels in 2021 (2021: 30.2%, 2019: 35.6%; *p* > 0.05). The 2021 puppies were less likely to be the only dog in the household (61.6%) than 2020 puppies (70.1%; *X*^2^ = 99.45, *p* < 0.001), returning to pre-pandemic (2019) levels (58.7%; *p* > 0.05). The majority of 2021 puppies lived in households with access to a private garden or yard (96.1%), which did not differ from 2020 (95.2%) or 2019 (96.9%) puppies (*X*^2^ = 11.94, *p* = 0.063).

#### 3.2.3. Impact of COVID on Household Lifestyle

The 2021 owners were less likely to work from home (51.0%) than 2020 owners (57.3%; *X*^2^ = 24.20, *p* < 0.001), returning to pre-pandemic (2019) levels (51.8%; *p* > 0.05). There was no difference between the proportion of 2021 owners who home-schooled their children (25.9%) compared with owners of 2020 puppies (27.5%), with the previously documented increase from 2019–2020 returning to pre-pandemic levels in 2021 (2019, 22.7%; *p* > 0.05). The proportion of 2021 owners who had been furloughed from their jobs (23.8%) did not differ from 2020 owners (24.5%, *p* > 0.05), remaining significantly lower than owners of 2019 puppies (30.6%; *X*^2^ = 18.72, *p* < 0.001). There was no difference in the proportion of owners who became unemployed due to COVID-19 between 2021 (4.9%), 2020 (5.8%) or 2019 (6.0%) owners (*X*^2^ = 0.36, *p* = 0.358). Owners of 2021 puppies were more likely to be keyworkers (42.3%) compared to 2020 (34.8%) and 2019 (33.7%) owners (*X*^2^ = 33.09, *p* < 0.001), but there was no difference in the proportion who had another household member considered to be a keyworker (2019: 24.8% vs. 2020: 24.3%; 2021: 24.6%; *X*^2^ = 0.10, *p* = 0.950).

### 3.3. Puppy Demographics

There was no significant difference between the sex distribution of 2021 puppies and 2020 or 2019 puppies (male, 2019: 51.7%; 2020: 53.4%; 2021: 53.8%; *X*^2^ = 1.13, *p* = 0.567). At the relative times of the survey for each year group, a significantly higher proportion of 2021 puppies had been neutered (24.5%) compared to 2020 puppies (19.5%; *X*^2^ = 250.54, *p* < 0.001). This proportion had not returned to 2019 levels (43.0%; *p* > 0.05); however, a significantly higher proportion of 2021 (43.4%) and 2020 (45.4%) puppy owners planned to neuter their dog in the future than 2019 puppy owners (21.4%; *X*^2^ = 397.19, *p* < 0.001). Insurance levels at the time of the survey did not significantly differ between 2021 puppies (84.5%) and either 2020 (83.9%) of 2019 (83.5%) puppies (*p* > 0.05). The proportion of 2021 owners who planned to insure their dog in the future (4.5%) had significantly reduced from 2020 (7.0%; *X*^2^ = 110.57, *p* < 0.001) and had returned to 2019 levels (3.2%; *p* > 0.05).

The proportion of puppies that were purebred in the 2021 population (70.9%) did not differ significantly from 2020 (70.3%; *p* > 0.05); however, levels had not returned to pre-pandemic (2019) levels (78.9%) and remained significantly lower. In parallel, the proportion of designer-crossbreed puppies (i.e., intentional crosses of purebred dog breeds such as the Cockapoo; Cocker Spaniel x Poodle) in the 2021 population (24.5%) did not differ significantly from 2020 (26.1%; *p* > 0.05) but had not returned to pre-pandemic (2019) levels, remaining significantly higher (18.8%; *X*^2^ = 32.70, *p* < 0.001). Between 2019 and 2021, the Cavapoo (Cavalier King Charles Spaniel x Poodle) rose from the 12th (1.7%) to the 7th (2.3%) most popular breed in the study population. The proportion of 2021 puppies registered with The Kennel Club (46.5%) did not differ significantly from 2020 (46.2%; *p* > 0.05); however, levels had not returned to pre-pandemic (2019) levels and remained significantly lower (58.2%; *X*^2^ = 69.39, *p* < 0.001). The 10 most popular dog breeds/crossbreeds among the puppies purchased for each year are described in Table 1.

### 3.4. Owner Demographics and Lifestyle: Multinomial Analysis

Multinomial regression modelling identified two variables related to owner demographics and lifestyle that had significant associations with year of acquisition (Table 2). Compared to 2019 owners, 2021 owners were less likely to have been furloughed during the pandemic but were more likely to have been a keyworker. 

### 3.5. Pre-Purchase Motivations

Companionship for the owner remained the most common reason cited as to why prospective owners wanted to purchase a dog across all three years (Table 3). The 2021 puppy owners were less likely to want to acquire a dog to encourage exercise, improve their family’s mental health, as companionship for other adults in their household, companionship for their children or for a specific working role, compared to Pandemic Puppy owners. These levels, however, had not yet returned to 2019 levels for improving their family’s mental health, with 2021 levels being significantly higher than 2019 (Table 3). The desire to acquire a dog for companionship for other dogs in the owners’ household remained significantly lower in 2021 than in 2019.

When deciding which breed or crossbreed to purchase, 2021 acquisition motivations varied widely compared to 2019 and Pandemic Puppy acquisitions. Although the most commonly cited characteristic sought by owners remained being a breed/crossbreed that was a good companion (most common reason cited across all three years) (Table 4), at the univariable level, 2021 owners were significantly less likely to seek a breed/crossbreed that was a good companion, whose size suited their lifestyle, that was generally healthy, good with children, easy to train, based on appearance, that would encourage exercise, or that was hypoallergenic compared to Pandemic Puppy owners. Most changes documented in 2020 had returned to 2019 levels; however, levels of 2021 owners seeking a puppy from a breed/crossbreed based on its working abilities (2019: 17.5%; 2020: 13.2%; 2021: 13.9%, *p* < 0.001) or that they had owned before (2019: 40.1%; 2020: 33.0%; 2021: 35.0%, *p* < 0.001) did not significantly differ from Pandemic Puppy levels, remaining significantly lower than 2019 levels.

Furthermore, some new changes in acquisition motivations were detected. Acquiring a breed/crossbreed based on appearance (2019: 39.3%; 2020: 39.7%; 2021: 34.6%, *p* < 0.001) or a breed/crossbreed being generally healthy (2019: 45.3%; 2020: 48.8%; 2021: 38.5%; *p* < 0.001) had both declined significantly below 2019 levels, having remained stable between 2019 and 2020. Furthermore, acquiring a breed based on its perceived long life expectancy had newly declined from 2019 (2019: 15.9%; 2020: 14.2%; 2021: 12.3%, *p* = 0.020). 

A breeder who would allow prospective owners to see the puppies’ mother (dam) was the most common characteristic owners sought out in a breeder across all three years (Table 5). At the univariable level, 2021 owners were significantly less likely to seek out a breeder they felt cared for their dogs or communicated well with them compared to Pandemic Puppy owners, both of which had returned to 2019 levels. In contrast, the likelihood of owners seeking a breeder that was a member of the Kennel Club Assured Breeders Scheme (2019: 25.2%; 2020: 20.2%; 2021: 18.9%, *p* < 0.001), or that bred from dogs that had been awarded prizes at dog shows (2019: 11.3%; 2020: 8.3%; 2021: 7.2%, *p* < 0.001) had not increased from 2020 levels, remaining significantly lower than 2019. The significant decline documented between 2019 to 2020 in owners seeking out a breeder that performed health tests for the breed/crossbreed they wanted continued to decline further in 2021 (2019: 66.9%; 2020: 62.1%; 2021: 58.0%, *p* < 0.001). Furthermore, some new changes in motivations for breeder characteristics were detected. For 2021 owners, seeking out a breeder who would allow the owner to see the puppies’ mother (2019: 90.5%; 2020: 88.9%; 2021: 86.1%, *p* < 0.001) or father (2019: 44.1%; 2020: 42.1%; 2021: 38.2%, *p* = 0.002), they felt was trustworthy (2019: 79.2%; 2020: 78.9%; 2021: 69.1%, *p* < 0.001), had the breed they wanted available (2019: 39.2%; 2020: 41.8%; 2021: 32.2%, *p* < 0.001), or had puppies available at the time they wanted (2019: 38.3%, 2020: 40.1%, 2021: 33.7%, *p* < 0.001) had significantly declined below 2019 levels, having remained stable between 2019 and 2020.

#### Pre-Purchase Motivations: Multinomial Analysis

Multinomial regression modelling identified ten variables related to pre-purchase motivations that had significant associations with year of acquisition, when compared to the 2019 pre-pandemic baseline (Table 6). Seven variables differed between 2021 and 2019 pre-purchase motivations at the multivariable level: 2021 puppy owners were more likely to acquire a dog to improve their/their families mental health, more likely to desire a breed/crossbreed that is good with children, less likely to desire a breed/crossbreed that is generally healthy, less likely to seek out a breeder that performed health tests, that would allow them to see their puppy’s mother or father, or was a member of the Kennel Club Assured Breeders Scheme (KC ABS).

### 3.6. Pre-Purchase Behaviours

**Figure 3:**
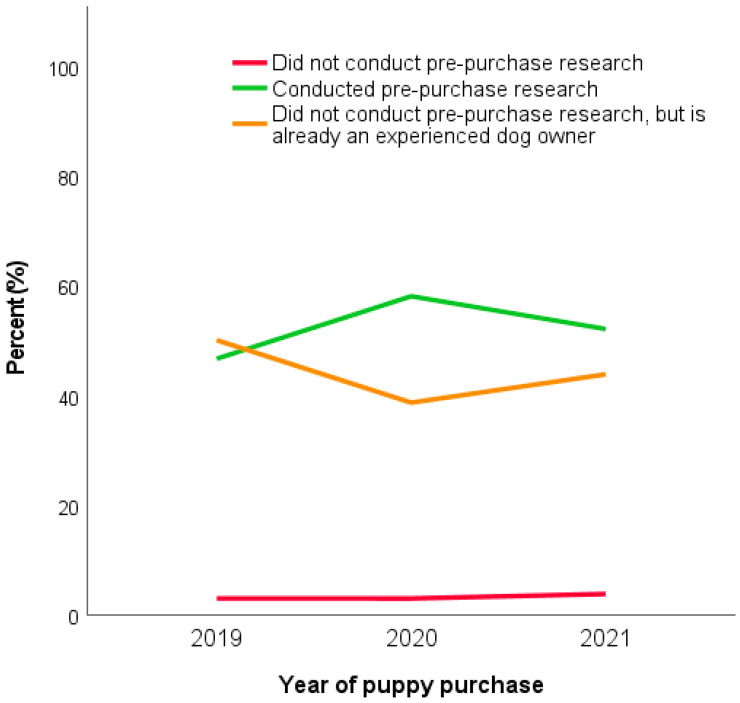
Engagement in pre-purchase research by 2019 puppy owners (*n* = 1148), 2020 Pandemic Puppy owners (*n* = 4355) and 2021 puppy owners (*n* = 1971) in the UK.

Pre-purchase research was carried out by significantly fewer 2021 owners (52.2%) compared to Pandemic Puppy owners (58.1%) but remained at significantly higher levels than 2019 (46.7%; *X*^2^ = 58.98, df = 2, *p* < 0.001) (Figure 3). This difference was largely attributable to the proportion of owners who considered themselves as an experienced dog owner and therefore felt they did not need to conduct pre-purchase research (2019: 50.3%, 2020: 38.9%, 2021: 43.9%) which followed the opposite pattern of decreasing in 2020, then increasing in 2021. Consistently few owners in all three cohorts reported that they conducted no pre-purchase research (2019: 3.0%, 2020: 3.0%, 2021: 3.9%), which did not significantly differ between years (*p* > 0.05).

Within the sub-population of owners who conducted pre-purchase research, the most common source of information across all three years was friends or family who own or had owned a dog. At the univariable level, 2021 owners were significantly less likely to conduct pre-purchase research by talking to friends and family who own/had owned a dog or by accessing a breed/crossbreed-specific online resource compared to Pandemic Puppy owners, both of which had returned to pre-pandemic 2019 owner levels. Levels of 2021 owners accessing animal charity websites had significantly declined compared to Pandemic Puppy owners but remained significantly higher than pre-pandemic 2019 owner levels. In contrast, levels of 2021 owners talking to a dog breeder had newly decreased compared to 2019 levels, and levels of 2021 owners accessing the Kennel Club website had significantly decreased compared to 2020 levels (Table 7).

Owners sourced their puppy most commonly via animal selling websites across all three years; however, at the univariable level, 2021 puppy owners were less likely to have found their puppy via animal selling websites than Pandemic Puppy owners, returning to pre-pandemic 2019 owner levels (Table 8). In contrast, the decrease in owners finding their puppy via a dog breeder they already knew or via The Kennel Club “Find a Puppy” search documented between 2019 and 2020 had persisted into 2021, with the level of 2021 owner finding puppies via these routes being significantly lower than 2019. Furthermore, the increase in owners finding puppies via recommendations from a friend between 2019 and 2020 had persisted into 2021, with the level of 2021 owners finding puppies via this route being significantly higher than 2019.

**Figure 4:**
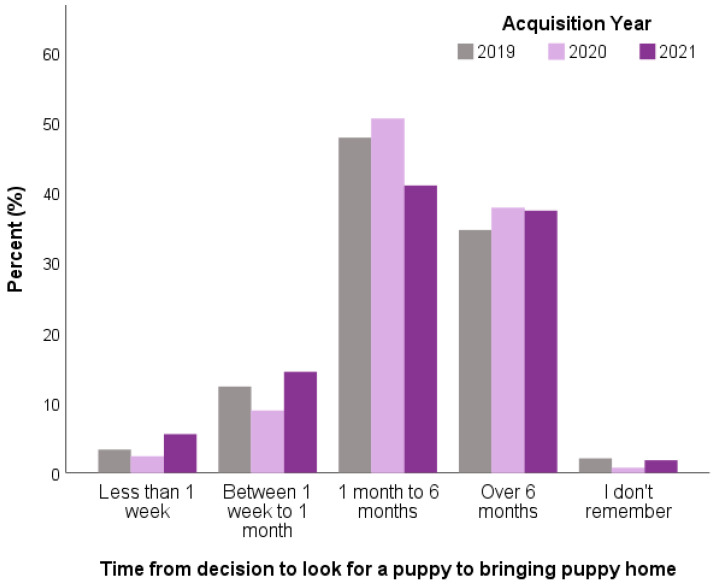
Interval from deciding to look for a puppy to acquisition (bringing puppy home) (*n* = 5401) with comparison between 2019 owners (*n* = 1124), Pandemic Puppy owners (*n* = 4277) and 2021 owners (*n* = 2080) in the UK.

In 2021, the interval from prospective owners’ initial decision to look for a puppy to when their puppy was brought home had shortened compared to previous years. The 2021 puppy owners were less likely to take 1 month–6 months between deciding to look for a puppy to when their puppy was brought home (41.9%) compared to 2020 owners (50.4%) or 2019 owners (47.8%), and conversely, were significantly more likely to take less than one week (5.5%) compared to 2020 owners (2.3%) or 2019 owners (3.3%) (47.6%; *X*^2^ = 132.43 *p* < 0.001) (Figure 4). Fewer than 1 in 4 of 2021 owners had joined a waiting list for their puppy (22.1%), a significantly lower proportion than in either 2020 (28.2%) or 2019 (26.8%; *X*^2^ = 21.84; *p* < 0.001).

**Figure 5:**
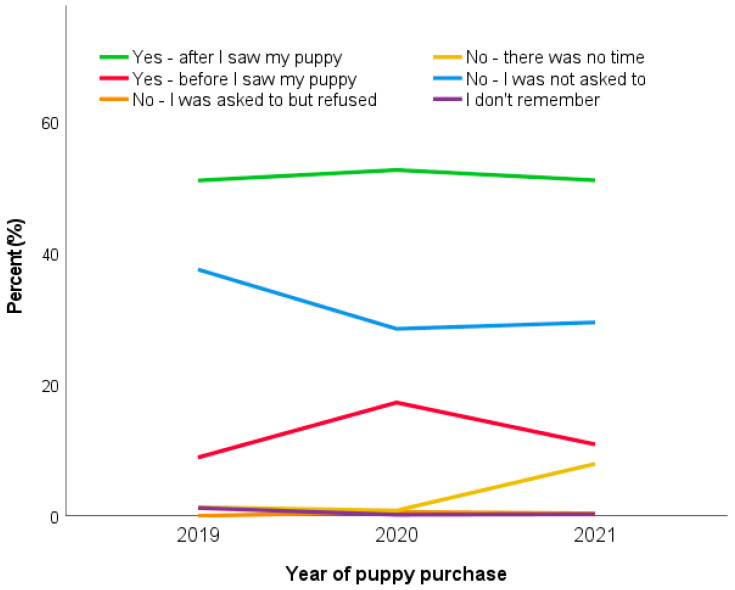
Deposit practices to secure puppies prior to purchase, with comparison between 2019 owners (*n* = 921), Pandemic Puppy owners (*n* = 3452) and 2021 owners (*n* = 2080) in the UK.

The 2021 puppy owners were less likely to put down a deposit before they saw their puppy (10.9%) compared with 2020 owners (17.2%; *X*^2^ = 316.79, *p* < 0.001), which had returned to 2019 levels (8.9%, *p* > 0.05). The proportion of owners who had not been asked to place a deposit for their puppy did not differ between 2021 owners (29.5%) and 2020 owners (28.6%) and remained significantly lower than 2019 levels (37.5%) (Figure 5).

The proportion of 2021 owners who visited what was reported to be their puppy’s breeder’s house in-person prior to the day they brought the puppy home had significantly increased compared to Pandemic Puppy owners (+10.6%); however, this had not recovered to pre-pandemic 2019 levels, remaining significantly lower (−10.4%; 2019: 80.6%; 2020: 59.6%; 2021: 70.2%; *X*^2^ = 193.23, *p* < 0.001).

**Figure 6:**
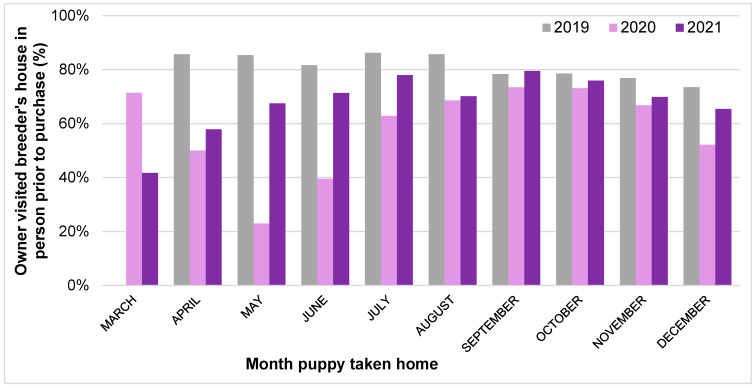
Percentage of puppies visited in person pre-purchase from March to December 2019–2021, depicted by year and month (2019 = 1081, 2020 = 4136, 2021 = 1845).

Multivariable binary logistic regression analysis explored the impact of acquisition year and month (and the interaction between these two variables) upon the likelihood of not viewing a puppy in person pre-purchase (Figure 6). Puppies taken home in March were significantly more likely to *not* be viewed in person pre-purchase compared to those taken home in December (OR: 2.65 (95% CI: 1.40–5.02), *p* = 0.003), while puppies taken home in April (OR: 0.47 (95% CI: 0.23–0.95), *p* = 0.035), May (OR: 0.48 (95% CI: 0.24–0.97), *p* = 0.043), July (OR: 0.45 (95% CI: 0.23–0.86), *p* = 0.016), or August (OR: 0.47 (95% CI: 0.26–0.85), *p* = 0.013) were less likely to *not* be viewed in person compared to those taken home in December. In four specific months, the likelihood of a puppy *not* being viewed in person prior to purchase were significantly higher than the corresponding month in 2019, three in 2020: April 2020 (OR: 2.32 (95% CI: 1.07–5.05), *p* = 0.034), May 2020 (OR: 7.59 (95% CI: 3.50–16.45), *p* < 0.001) and June 2020 (OR: 2.63 (95% CI: 1.32–5.24), *p* = 0.006), and one in 2021: April 2021 (OR: 2.95 (95% CI: 1.29–6.74), *p* = 0.01). In contrast, two specific months had a lower likelihood of a puppy *not* being viewed in person prior to purchase than in 2019, both in 2020: September 2020 (OR: 0.51 (95% CI: 0.27–0.95), *p* = 0.036), and October 2020 (OR: 0.52 (95% CI: 0.28–0.96), *p* = 0.04).

Corresponding to the decrease in puppies being viewed in person pre-purchase, there was a significant decrease in 2021 owners who saw what was claimed to be their puppy via photos/pre-recorded videos (−12.0%; 2019: 31.3%; 2020: 51.5%; 2021: 18.2%; *X*^2^ = 273.58, *p* < 0.001) or via live video calls (−10.7%); however, neither of these practices had returned to pre-pandemic 2019 levels, remaining significantly higher. The proportion of owners who did not ask to view their puppy at all significantly decreased in 2021 compared to both 2019 and 2020 (Table 9).

The number of owner visits to see their puppy in person prior to being brought home remained significantly lower in 2020 and 2021 than pre-pandemic levels, with the median (25th–75th centile) visits for 2021 owners being 1 (1–2), remaining significantly lower than pre-pandemic 2019 levels (2019: 2 (1–2); 2020: 1 (1–2); *p* = 0.03). Correspondingly, the median (25th–75th centile) number of live video calls with the breeder of 2021 puppies was significantly higher at 1 (0–2), compared to either 2020 (0; 0–3; *p* < 0.001) or 2019 (0; 0–0, *p* < 0.001).

Significantly more 2021 owners were questioned on their suitability as a dog owner before their breeder agreed to sell them their puppy (79.1%) compared with 2020 owners (75.3%) or 2019 owners (75.5 %; *X*^2^ = 20.71, *p* < 0.001). One in five 2021 owners did not purchase their first-choice of breed (first-choice: 80.0%) and were less likely to purchase their first-choice compared to 2019 or 2020 dog owners (2019: 91.6%; 2020: 86.6%; *X*^2^ = 228.78, *p* < 0.001) (Table 10). 

#### Pre-Purchase Behaviours: Multinomial Analysis

Multinomial regression modelling identified seven variables related to pre-purchase behaviours that had significant associations with year of acquisition when compared to the 2019 pre-pandemic baseline (Table 11). Five variables differed between 2021 and 2019 pre-purchase behaviours. Compared to 2019 owners, 2021 were more likely to conduct pre-purchase research using charity websites, were less likely to find their breeder via The Kennel Club website or to visit their breeder in person prior to purchase and were more likely to view their puppy via video recordings and/or photographs or via live video calls.

### 3.7. Purchase Behaviours

The previously documented decrease in puppies collected from inside their breeder’s property between 2019 and 2020 (−33.7%) had not yet recovered to 2019 levels, remaining significantly lower in 2021 (−17.5%; *X*^2^ = 464.07, *p* < 0.001), with around 1 in 3 dogs still not being collected from inside their breeder’s property in 2021. Correspondingly, the previously documented increase in puppies collected from outside their breeder’s property between 2019 and 2020 (+24.3%) had not yet recovered to 2019 levels, remaining significantly higher in 2021 (+8.2%; *X*^2^ = 399.36, *p* < 0.001).

**Figure 7:**
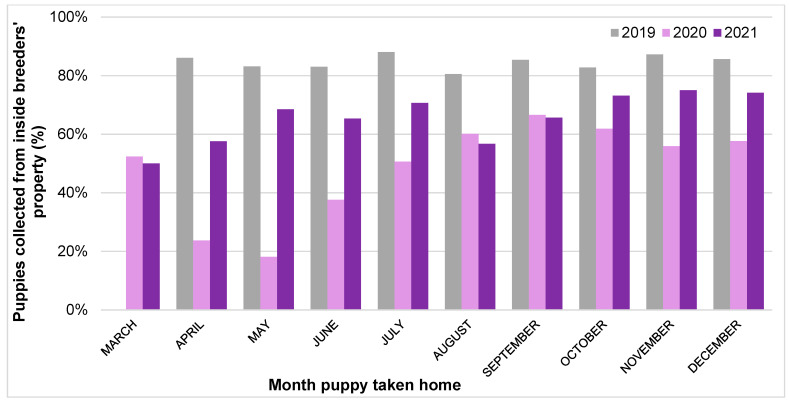
Percentage of puppies collected from inside their breeders’ property from March to December 2019–2021, depicted by year and month (2019 = 1099, 2020 = 4180, 2021 = 1860).

Multivariable binary logistic regression analysis explored the impact of acquisition year and month (and the interaction between these two variables) upon the likelihood of *not* collecting a puppy from inside the breeder’s property (Figure 7). Puppies taken home in March were significantly more likely to *not* be collected from inside their breeder’s property compared to those taken home in December (OR: 2.87 (95% CI: 1.52–5.41), *p* = 0.001), and puppies taken home in 2020 (OR: 4.42 (95% CI: 2.70–7.24), *p* < 0.001) or 2021 (OR: 2.09 (95% CI: 1.21–3.61), *p* = 0.008) were more likely to *not* be collected inside their breeder’s property compared to those taken home in 2019. In two specific months, the likelihood of a puppy *not* being collected from inside their breeder’s property was significantly higher than the corresponding month in 2019: April 2020 (OR: 4.64 (95% CI: 2.01–10.72), *p* < 0.001) and May 2020 (OR: 5.02 (95% CI: 2.26–11.17), *p* < 0.001).

The previously documented increase in rarer puppy collection locations between 2019 and 2020, including deliveries straight to their owners’ property or collection from a service station or car park, had returned to pre-pandemic (2019) levels in 2021 (*p* > 0.05) (Table 12).

The previously documented increase between 2019 and 2020 in owners *not* asking their breeder to see any information related to the health testing for to their puppy’s parents had reduced for DNA (genetic) tests, returning to 2019 levels (*p* > 0.05). In contrast, the increase in owners *not* asking for the results of veterinary screening tests (e.g., hips, elbows, knees, eyes, or respiratory testing) had reduced in 2021 (from an additional +10.4% not asking in 2020, to an additional +6.6% not asking in 2021) but remained significantly higher than 2019 (*p* < 0.001) (Table 13).

The previously documented decrease in puppies seen with their mother on the day they were acquired between 2019 and 2020 (−10.6%) had recovered to 2019 levels in 2021 (−0.8%, *p* > 0.05), as had the previous decrease in puppies seen with their littermates in the same period (−12.8%), which had recovered to 2019 levels in 2021 (−3.3%, *p* > 0.05) (Table 14). Correspondingly, the previous +7.8% increase in puppies seen on their own between 2019 (4.9%) and 2020 (12.7%) had returned to 2019 levels in 2021 (+1.4%; 6.3%, *X*^2^ = 93.97, *p* < 0.001).

Around one in seven breeders of puppies purchased in 2021 had another litter for sale at the same time, most commonly of the same breed/crossbreed (9.2%) or of a different breed/crossbreed (4.4%); these proportions did not significantly differ from 2019 (8.7% and 4.6%, respectively) or 2020 (10.1% and 4.4%, respectively; *X*^2^ = 14.70, *p* = 0.065). Very few 2021 owners felt pressured by their puppy’s breeder to commit to purchasing their puppy (1.6%), which did not differ from 2020 (2.3%) or 2019 (1.5%; *X*^2^ = 7.07, *p* = 0.314).

At time of acquisition, 54.5% (*n* = 997) of 2021 puppy owners were told by their puppy’s breeder that their puppy was 8 weeks old at sale, followed by 15.6% (*n* = 285) that were told their puppy was 9 weeks old. A minority of dogs (4.0%) were sold under 8 weeks of age in 2021. When comparing to previous years, 57.6% of 2021 puppies were 7–8 weeks old at the time of sale, which was significantly lower than 2020 puppies (67.3%) but had not yet returned to pre-pandemic (2019) levels (52.5%; *X*^2^ = 171.61, *p* < 0.001). In contrast, the proportion of puppies aged 11 to 12 weeks had significantly increased in 2021 (11.6%) from 2020 levels (5.5%) and had returned to pre-pandemic (2019) levels (11.5%; *p* > 0.05).

A significantly greater proportion of 2021 puppies were purchased with a pet passport (10.4%) than either 2020 (7.1%) or 2019 (4.1%; *X*^2^ = 75.34, *p* < 0.001). The age (in weeks) of puppies sold with a passport significantly differed between years of acquisition (*X*^2^ = 20.71, *p* = 0.008) (Table 15). The most common age at acquisition for puppies sold with a pet passport continued to be 7–8 weeks (2019: 36.1%, 2020: 56.5%, 2021: 49.7%). The proportion of puppies sold over the age of 13 weeks continued to decline in 2021 (−17.1% compared to 2019), with 89.3% of puppies sold with a passport aged under 13 weeks in 2021, compared to 87.4% in 2020 and 72.2% in 2019.

**Figure 8:**
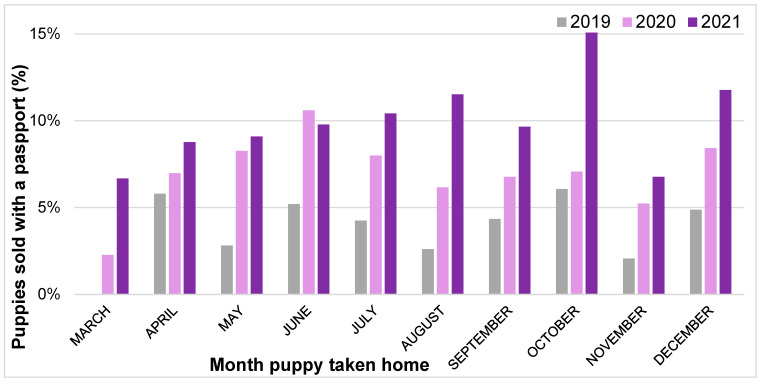
Percentage of puppies sold with a passport from March to December 2019–2021, depicted by year and month (2019 = 1150, 2020 = 4398, 2021 = 2080).

Multivariable binary logistic regression analysis explored the impact of acquisition year and month (and the interaction between these two variables) upon the likelihood of a puppy being sold with a passport (Figure 8). Puppies taken home in 2021 were significantly more likely to be sold with a passport than those taken home in 2019 (OR: 2.62 (95% CI: 1.03–6.66), *p* = 0.043). No effects of specific months, or interactions between month and year were identified (*p* > 0.05).

**Figure 9:**
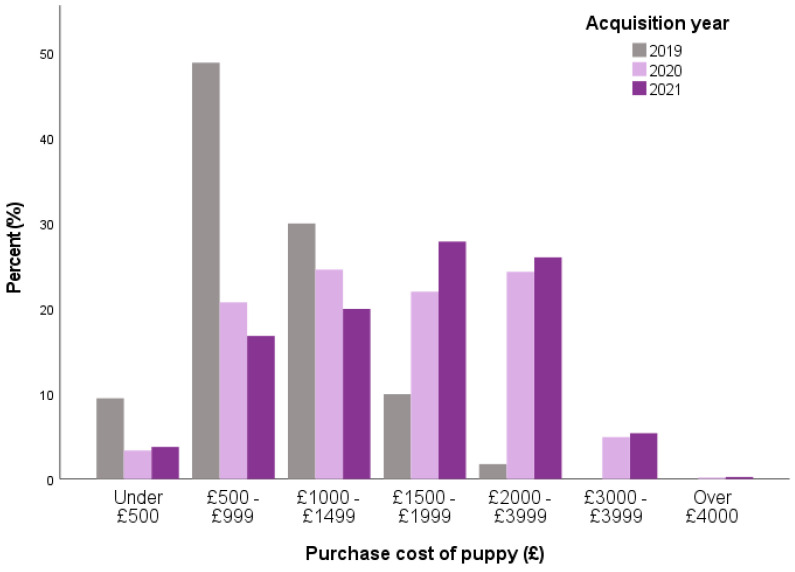
Purchase price of puppies, excluding any associated purchases such as food, collar, bowls, etc., with comparison between 2019 owners (*n* = 1022), Pandemic Puppy owners (*n* = 3835) and 2021 puppies (*n* = 2080) in the UK.

The significant rise in puppy price documented between 2019 and 2020 persisted into 2021, with over one quarter of 2021 puppies sold for £2000–£2999 (26.0%), with the proportion of puppies sold for this price not differing from 2020 (24.3%) and remaining significantly more common than in 2019 (1.8%; *X*^2^ = 780.33, *p* < 0.001) (Figure 9).

Levels of awareness of The Puppy Contract prior to purchase did not differ between years of acquisition (2019: 41.5%; 2020: 40.2%; 2021: 38.7%; *X*^2^ = 2.23, *p* = 0.329). Within the population of owners who were aware of The Puppy Contract pre-purchase (*n* = 2540), the level of use significantly declined in 2021 compared with previous years (2019: 63.0%; 2021: 62.8%; 2021: 57.2%; *X*^2^ = 6.97, *p* = 0.031). The most common reason for not using (either choosing not to use or being unable to use) The Puppy Contract continued to be the belief that it was not needed as the owners were confident in their own purchasing decision, levels of which had significantly increased (+14.4%) in 2021 compared to 2019 (*X*^2^ = 10.79, *p* = 0.005). Similarly, the proportion of owners who forgot to use it when purchasing their puppy had significantly increased (+6.6%) in 2021 compared to 2019 (*X*^2^ = 7.19, *p* = 0.027) (Table 16). Several other reasons that 2021 puppy owners did not use the contract had significantly increased compared to 2020 Pandemic Puppy owners, including using a different written contract (+7.3%), using a different verbal contract (+4.8%), not feeling it was needed as the breeder was someone they knew (+9.5%), and not knowing enough or feeling confident enough to use it (+5.3%) (Table 16).

#### Purchasing Behaviours: Multinomial Analysis

Multinomial regression modelling identified twenty variables related to purchasing behaviours that had significant associations with year of acquisition when compared to the 2019 pre-pandemic baseline (Table 17). Seven variables differed between 2021 and 2019 purchase behaviours. Compared to 2019 owners, 2021 were less likely to collect their puppy from inside their breeder’s property, instead being more likely to collect from outside their breeder’s property; were less likely to receive a health test (veterinary screening information) having asked for it; and were less likely to purchase a puppy priced <£2000 (<£500, £500–999, £1000–1499; £1500–1999).

### 3.8. Influence of COVID on Puppy-Purchasing Decisions in 2021

Of those households that purchased puppies during 2021, 17.5% had not considered purchasing a puppy before the COVID-19 pandemic, significantly higher than 2020 Pandemic Puppy owners (11.0%; *X*^2^ = 59.08, *p* < 0.001). Despite this, there was a significant reduction (−19.5%) in owners who felt their decision to purchase a puppy had been influenced by the COVID-19 pandemic in 2021 (22.0%) compared to 2020 (41.5%; *X*^2^ = 210.43, *p* < 0.001). Of those 2021 owners who felt the pandemic had influenced their decision (*n* = 383), the most common reason was having more time to care for a dog (76.8%); however, this reason had significantly reduced (−9.9%) compared to 2020 Pandemic Puppy owners (86.7%, *X*^2^ = 23.73, *p* < 0.001). Similarly, boredom due to lockdown restrictions had significantly reduced as a purchase influence between 2020 and 2021 (−2.5%; *X*^2^ = 5.27, *p* < 0.022) (Table 18).

## 4. Discussion

This study has charted both the recovery and persistence of various pandemic-related changes to UK puppy-purchasing motivations and behaviours in 2021. While the majority of changes documented in 2020 had returned to their 2019 pre-pandemic baseline, others have continued into 2021. Of high concern, is that some peak-pandemic-related changes have exacerbated beyond 2020 levels in 2021. If these ongoing changes were not linked to lockdown restrictions that have now been lifted, they risk becoming longer-term features of UK puppy-purchasing culture.

### 4.1. Continued Rise in Puppy Imports

The significant increase in puppies sold with a passport in 2020 compared to 2019 did not normalize to pre-pandemic levels in 2021, instead, significantly increasing beyond peak-pandemic 2020 levels and more than doubling since 2019. More than 1 in 10 puppies purchased in 2021 were sold with a passport, with 2021 puppies at a 2.62 increased odds of being sold with a passport than 2019 puppies. An increasing number of these puppies were sold with a passport under the age of 13 weeks in 2021 compared to 2019 and 2020. Given that the minimum age at import under both the Pet Travel Regulations [46] and the Balai Directive [47] is 15 weeks, this likely indicates an increase in illegal importations of puppies to the UK over this three-year period. When examining the month-by-month analysis of puppies sold with passports over this period, peaks were not associated with periods of lockdown restrictions; for example, the two months with the highest levels of puppies sold with passports in 2021 were in the autumn–winter, when all restrictions on movement had been lifted [48]. This may reflect a concerning trend towards the UK puppy market becoming increasingly reliant upon the supply of imported puppies, even in the face of reports that overall demand for dogs is dropping compared to peak-pandemic, and thus importation is not simply a reflection of markedly increased demand [49]. These results are in line with reports from UK animal charities, e.g., RSPCA reported an 11% increase in commercially imported dogs from 2020 to 2021 [50].

With the current lack of progress with the Kept Animals Bill [51], which following calls from charities such as Dogs Trust proposed to raise the minimum import age for dogs coming into the UK to six months to reduce the desirability of puppies [52], alongside insufficient penalties for illegally importing puppies, there is a reliance upon changing consumer behaviour to tackle this concerning trend of high importation at present. However, RSPCA research suggests that 38% of people would buy a dog smuggled into the UK from another country, with suggestions these people may think they are helping rescue an animal from poor conditions or are unaware of the hidden risks [50]. Work is urgently needed to gauge owner awareness of what a puppy offered for sale with a passport represents, e.g., whether they know this means the puppy has been imported (and the welfare and public health implications of this), and whether they understand key indicators of a legal vs. an illegal sale. From this, effective human behaviour change interventions are required to reverse this trend, to reduce and halt the importation of underage puppies, often via organized crime groups [53], becoming an accepted supply source within the UK market and the subsequent impacts on canine welfare both within and outside the UK [53]. This is additionally pertinent considering growing evidence of infectious disease risks of imported puppies to public health and the health of dog’s bred and resident in the UK (e.g., [54,55]). Given the significant increase in imported dogs, animal health professionals who interact with puppies should be increasingly aware and vigilant for indicators of illegal importation and prepared to raise suspicions to appropriate authorities [56].

This ongoing shift towards imported puppies also raises a wider question around how demand can be met by breeding within the UK alone going forwards, given reports that there was already a shortage of legitimate breeders to reach UK demand, even pre-pandemic [57]. Finding ways to upscale legal, domestic breeding of puppies is likely needed to fill this puppy gap, although progress here may face barriers given the ongoing stigma and preconceptions of ‘hobby’ or ‘backyard’ breeding in the UK. Outdated perceptions of dog overpopulation and lack of awareness of dog importation in the US have reshaped their dog market towards imported rescues, which has been influential in the decline of local dog breeding [58]. Exploring how UK consumers perceive the current structure of the UK dog market, including the scale of different sources (e.g., estimates of the number of available dogs/year from rescue organizations, or from high-welfare accredited breeders), would be valuable to aid understanding of a prospective owners’ perceived likelihood of purchasing from different sources (and subsequent measures to reduce risk if acquiring from certain sources), and if required, shifting understanding towards the need for a greater number of domestic breeders to avoid the welfare harms of importation.

### 4.2. Ongoing Impacts on Pre-Purchase Viewing and Puppy Collection Behaviours

The level of owners carrying out recommended pre-purchase viewing and purchase collection behaviours remained reduced during 2021, with 2021 buyers being less likely to view their puppy in person prior to purchase or to collect their puppy from inside their breeder’s property compared to pre-pandemic 2019 levels. Instead, 2021 owners remained more likely to view their puppy via live video calls, video recordings and/or photographs, and to collect their puppy from outside their breeder’s property, without normalization to 2019 pre-pandemic levels. Some of these persistent changes are likely to be related to the ongoing limits to social interaction and movement in 2021 still imposed by the Government during this phase of the pandemic, including the third national lockdown from January 2021 to March 2021, followed by a phased exit from lockdown until July 2021 [48]. This is reflected in the month-by-month analysis of the proportion of pre-purchase in-person visits, and collection of puppies from inside breeders’ properties. These were lowest in early 2021 compared to later that year, likely due to prospective owners adhering to ‘stay at home’ orders (which continued until 29 March 2021). Given the vital importance of both of these purchasing behaviours in reducing the likelihood of purchasing from a low welfare, illegal breeder or dealer who is likely to try to conceal the origins of the puppies they sell [59], the persistent reduction in these behaviours between 2020–2021 should be monitored to ensure that they return to pre-pandemic levels (e.g., in 2022, when no pandemic-related restrictions to the public’s movements were imposed). Ideally, they would increase beyond these levels, given they were still not 100% (even when collection from their place of birth is a legal requirement for breeders). Making the public aware that although potentially appealing, a convenience culture of puppies being readily viewed online and then purchased at first visit is likely to be detrimental to canine welfare, even with human lives (and particularly purchases) being increasingly based online in the ‘post-pandemic’ era. With animal-selling websites being the most common place owners sourced their puppy across all three years of this study, increasing consumer literacy around responsible online puppy purchasing that maximizes canine welfare [60], as well as increased scrutiny of [61], and corporate social responsibility activities from online sellers, is likely to be needed.

### 4.3. Impact of COVID-19 on the Likelihood of and Reasons for Acquiring a Puppy

The COVID-19 pandemic appears to have had a persistent influence upon households choosing to buy a puppy, with a significant increase in households purchasing puppies who had not considered purchasing a puppy before the pandemic compared to 2020 owners. This may simply reflect the increased time these households would have had to make this decision since the start of the pandemic compared to 2020 owners; however, it could also reflect exposure to the ongoing media focus upon dog ownership and puppy purchasing, e.g., stories on the positive impact of dog ownership during the pandemic [62,63] and purported ‘shortages’ of puppies in 2020 [64]. This could also reflect lifestyle changes during 2021 that resulted in household routines better suited to dog ownership. For example, 2021 puppy owners were more likely to have been keyworkers compared to 2020 or 2019 owners, which may have precluded those households from acquiring puppies during the peak periods of the pandemic where their services were most needed [65] and thus delayed this consideration. Despite this, a significantly lower proportion of owners reported that they felt their decision to buy a puppy had been specifically influenced by the COVID-19 pandemic compared to 2020 owners, almost halving from around 2 in 5 owners to 1 in 5 owners. Within this, increased time to care for a dog remained the most common reason that the pandemic had influenced their decision (over three-quarters of owners); however, this had significantly reduced from 2020, likely reflecting the post-peak pandemic lifestyle changes experienced by many UK residents, e.g., returning to full or partial working away from the home [66]. Similarly, although a rare influence across both years studied, boredom due to lockdown restrictions had significantly reduced as a purchase influence between 2020 and 2021, again likely due to the larger proportion of 2021 spent outside of lockdown compared to 2020 [48]. Free-time as an acquisition motivator, and the potential for it to change with household circumstances remains a risk factor for relinquishment [67], and indeed, recent evidence suggests that pets acquired during the pandemic were twice as likely to be considered for, or be, given up compared to those acquired > 6 months before the pandemic [68]. This is likely to be exacerbated with the current cost-of-living crisis, with reports of increased relinquishment due to owners’ increasing financial constraints [69].

### 4.4. Ongoing Impact on Puppy Prices

Often grabbing media headlines during the pandemic [70], the rise in the price of puppies during 2020 persisted into 2021, with more than 1 in 4 2021 puppies being sold for over £2000, remaining significantly more common than pre-pandemic (less than 1 in 50 puppies). A similar increase in prices was reported by Dogs Trust from the National Dog Survey, with buyers paying over £2001 only becoming prevalent in 2020 and 2021 (compared to 2011–2019), with buyers paying £1001–2000 also notably increased in this period [13]. Online seller Pets4Homes noted a persistent increase in puppy prices in 2021, with the average sale price for dogs more than doubling (×2.5) in March 2021 compared to pre-pandemic (February 2020) [49]. This persistent increase raises several welfare concerns, including the encouragement of unscrupulous breeders and dealers to meet this lucrative demand through low-welfare breeding and rearing, and in light of the current cost-of-living crisis, leaves owners with less money remaining for the ongoing care of their dog (e.g., regular feeding and husbandry costs, but also potentially unforeseen veterinary costs) [71]. To encourage changes in buyer behaviour, increasing buyer awareness that high prices do not necessarily indicate the high quality of a breeder or indeed of the dog are needed to dispel misconceptions and to highlight that their money is potentially funding organized crime gangs within the illegal puppy market, with the market for illegally traded puppies believed to be worth £13 million [72].

### 4.5. Changes to Dog Demographics

An ongoing demographic shift away from registered pedigree dogs and, more broadly, purebred dogs, was documented in several areas of the 2021 study. A persistent reduction in owners seeking out a breeder that was a member of the Kennel Club Assured Breeders Scheme was seen in 2021, remaining significantly lower than 2019 levels (from 1 in 4 to less than 1 in 5 owners). This likely reflects a wider shift away from Kennel Club registered dogs, with a persistent reduction in owners finding their puppy’s breeder via The Kennel Club website, down from 1 in 6 owners in 2019 to 1 in 10 owners in 2021. This likely influenced the proportion of puppies registered with The Kennel Club remaining persistently lower in 2021 (−11.7%) than pre-pandemic 2019 levels. Outside of pedigree registration systems, the previously documented peak-pandemic decrease in purebred (registered *or* unregistered) puppies persisted into 2021, remaining significantly lower than pre-pandemic 2019 levels. In parallel, the increase in designer-crossbreed puppies documented in 2020 persisted into 2021, remaining significantly higher than pre-pandemic (from around 1 in 5 in 2019 to 1 in 4 in 2020 and 2021). The most marked shift in popularity of an individual designer-crossbreed was the Cavapoo (Cavalier King Charles Spaniel x Poodle, often Miniature or Toy), which rose from the 12th to the 7th most popular breed in the study population between 2019 and 2021. This echoes wider industry trends, with animal-selling website Pets4Homes reporting that the Cavapoo attracted the most buyers per dog listed in 2020 and 2021, attracting 283 buyers/dog advertised in June 2021 compared to a peak of 590 buyers/dog during 2020 and a low of 91 buyers/dog pre-pandemic [49]. Initial data from our team indicates concerning disparities between the motivations for acquiring a designer-crossbreed (e.g., perceived hypoallergenicity) and reality (e.g., lack of evidence of hypoallergenicity in designer-crossbreeds [73]), which may lead to owner dissatisfaction and increase relinquishment risk [74]. However, understanding the wider welfare implications of this shift requires further investigation, including study of the behavioural and health profiles of designer-crossbreeds compared to their progenitor purebred breeds, to assess whether this is a net positive or negative shift at the overall dog population level.

### 4.6. Impacts on Motivators for Acquiring a Dog, and Specific Breeds/Crossbreeds

#### 4.6.1. Ongoing Desire for Mental Health Support

Although many motivators for dog acquisition that peaked during the pandemic had returned to pre-pandemic levels (e.g., exercise encouragement, companionship for children), the desire to acquire a dog to improve household mental health had persisted into 2021, remaining significantly higher than in 2019. This concurs with evidence of ongoing negative impacts of the pandemic upon mental health in the UK that may have driven this desire. A longitudinal study of UK adults (*n* = 2691) explored mental health throughout the pandemic, from the first wave (March 2020) to the seventh wave (February 2021) of COVID-19, and found that mental health problems were not restricted to the early pandemic and instead persisted into 2021 [75]. For example, depressive symptoms and loneliness increased from October 2020 to February 2021 (waves 6 to 7), and feelings of defeat and entrapment increased from July 2020 (wave 5) to October 2020 (wave 6), with this increase persisting into February 2021 (wave 7) [75]. This is also reflected in the demand for mental health services; for example, in 2021–2022, there was an increase in referrals for talking therapy (for common mental health problems such as depression and anxiety) compared to 2019–2020, going from 1.69 to 1.81 million referrals [76]. Although some studies have found evidence of improvement in mental health symptoms in pet owners during the pandemic, results are mixed (e.g., [77,78,79,80]). For example, in a US study, positive effects were not consistent across all owners and differed based on individuals’ level of attachment to their pet and their own psychological symptoms [81]. As such, caution should be exercised when recommending pet and/or dog ownership as a solution to mental health concerns, particularly for those with severe symptoms.

#### 4.6.2. Shift Away from Demand for ‘Easy’ Dogs

The shift documented during 2020 towards owners seeking out a breed/crossbreed perceived to be ‘easy’ and would fit in with an owners’ existing lifestyle appeared to have subsided in 2021. This was reflected in the level of owners seeking a breed/crossbreed considered easy to train, hypoallergenic or whose size suited their lifestyle had all returned to pre-pandemic levels. This may reflect the demographic shift back to more experienced dog owners acquiring puppies in 2021, with levels of previous dog ownership significantly increased from 2020, returning to pre-pandemic levels. Additionally, 2021 owners were more likely to work in the canine and/or animal care sector than 2020 owners, again returning to pre-pandemic levels. The latter may reflect waning concerns over contributing to the negative aspects of the ‘Pandemic Puppy’ phenomenon that was widely discussed within the animal welfare sector internationally during 2020, e.g., supporting unscrupulous breeders [82]. In addition, the desire to acquire a dog to encourage exercise returned to pre-pandemic levels, which was twinned with normalization of levels of owners seeking a breed/crossbreed that they perceived would encourage exercise. This may reflect changes in lifestyle between 2020 to 2021, with less time spent under strict lockdown restrictions which had limited activity outside of the households to daily walks during some periods of 2020 [48], likely precipitating some households to acquire puppies for this purpose.

#### 4.6.3. Ongoing Desire for ‘Child Safe’ Breeds and Dog Types

The desire for a breed/crossbreed that was good with children remained higher in 2021 than pre-pandemic; however, 2021 puppies were less likely to live in a household with children than 2020 owners, returning to 2019 levels. Some of this ongoing focus upon the perceived suitability of a breed/crossbreed with children may be related to the ongoing media focus on pediatric dog bites during the pandemic, with headlines such as “*Dog-bite Britain: the problem with the Pandemic puppy explosion*” [83] and “*Dog Bites Are On The Rise: What Parents Need To Know*” [84], often reporting on international studies of dog bite statistics during the pandemic [85,86,87]. Although increased caregiver focus on preventing dog bites to children is positive for public health, assuming that ‘safety’ will be conferred by certain breeds/crossbreeds is likely an ineffective strategy in isolation, given that any dog can bite, regardless of breed, and all child–dog interactions must be highly supervised [88].

#### 4.6.4. New Decline in Desire for a Healthy Dog

The desire to acquire a healthy dog appeared to decline in 2021 compared to both pre-pandemic (2019) and peak-pandemic (2020) periods. During the pre-purchase process, 2021 owners were less likely to desire a breed/crossbreed that is generally healthy, with fewer than 2 in 5 owners seeking out a breed/crossbreed based on this characteristic. This represented a significant −10.3% decline below 2020 levels, having remained stable between 2019 and 2020. This was further reflected in decision-making over which breeder to purchase from, with 2021 owners being less likely to seek out a breeder that performed relevant health tests on their breeding dogs, with a year-on-year decline between 2019 and 2021. Further interrogation of why health is a declining priority for puppy buyers, and which other factors are prioritized above it [26], is of key importance to ensure that sufficient pressure is placed upon breeders by buyers to supply puppies with good health and maximize dog health at the population level.

#### 4.6.5. New Decline in Desire to See Puppies’ Parents

Despite a breeder allowing a buyer to see their puppy’s mother being the most common desirable characteristic in a breeder across all three years studied, a new decline in motivation to find a breeder that would allow the owner to see either their puppy’s mother or father was documented in 2021, having remained stable between 2019 and 2020. Although the level of owners who saw what was purported to be their puppy’s mother had returned back to pre-pandemic levels in 2021 following a −10% decline during the peak-pandemic period, >15% still did not achieve this, mirroring the 14% of owners who did not prioritize this being offered by their puppies’ breeder in 2021. Further awareness is needed to make prospective owners aware of the legal requirement for puppies to be sold with their mother [28], and the reasons behind this. If the risk of acquiring a puppy from an illegal, low-welfare breeder (which a puppy being sold without its mother is likely a strong indicator of) is insufficient to change buyer behaviour, then awareness of the increased risk of future behavioural problems in puppies sold without their mother may be more effective given the greater emphasis of the individual impact upon the owner [89].

## 5. Conclusions

Although some of the most concerning changes to puppy purchasing induced by the peak-phase of the COVID-19 pandemic (e.g., dramatic reduction in puppies seen with their mother) had returned to pre-pandemic levels in 2021, several worrying puppy-purchasing trends persisted into 2021 that require further monitoring and human behaviour change interventions to tackle going forward. These could include continued efforts to raise awareness and change the behaviour of consumers (prospective puppy buyers), but also potentially legislative approaches to prevent the greatest harms, particularly those that are challenging to prevent by shifting consumer demand alone, or where these behaviours appear intractable. The continued rise in puppies being sold with passports, even beyond the peak phase of demand for puppies during the pandemic, indicates a concerning shift in the supply of puppies to the UK market and requires urgent measures to avoid associated welfare harms upon the puppies themselves, but also their dams outside of the UK and public health, given infectious disease risks. Although this study reports many pre-purchase and purchase behaviours returning to their pre-pandemic levels, many buyers in both 2019 and 2021 did not adhere to best practice when purchasing their puppy, leaving themselves vulnerable to purchasing from poor-welfare sources. Given the critical importance of both breeding and early life experiences upon future health and behaviour, efforts to improve buyer behaviour are still urgently needed to safeguard the welfare of future generations of UK dogs.

## Figures and Tables

**Figure 1:**
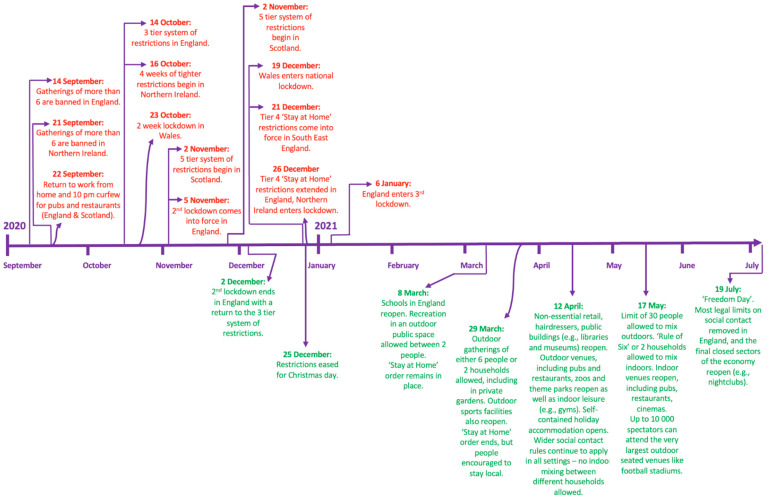
Timeline of COVID-19 restrictions in the UK from autumn 2020 to July 2021, when most legal limits to social contact were removed in England. The events shown here are not exhaustive but serve to demonstrate the main periods of social restrictions upon residents of the UK from September 2020, the earliest that puppies studied in the present study could have been born to be purchased from 16 weeks of age in 2021.

**Table 1:** Most popular individual breeds/designer-crossbreeds in the 2019 puppy (*n* = 1147), 2020 Pandemic Puppy (*n* = 4358) and 2021 puppy (*n* = 2080) cohorts. Crossbred dogs were defined as mixes of breeds of unknown origin, or crosses who were not reported using a breed-indicative designer-crossbreed name, e.g., Spaniel Cross. The breeds with the greatest frequency, which formed the top ten most popular breeds for each of the 2019 puppy, Pandemic Puppy and 2021 cohorts are shown along with their ranking (1 = most popular) for each year. The *p* value reflects the probability of differences in popularity for the breed/crossbreed between 2019 and 2021. Significant differences in breed/crossbreed population % between 2019 and 2020 or 2020 and 2021 are emboldened. NS = Non-significant (*p* > 0.05).

Breed/Crossbreed	2019			2020			2021			Statistics			
	*n*	%	Rank	*n*	%	Rank	*n*	%	Rank	% Change 2019 to 2021	Sig	% Change 2020 to 2021	Sig
**Labrador Retriever**	**134**	**11.7**	**1**	**429**	**9.9**	**1**	**271**	**13.0**	**1**	**+11.11%**	**NS**	**+31.31%**	**<0.05**
**Cocker Spaniel**	**81**	**7.1**	**2**	**324**	**7.4**	**3**	**202**	**9.7**	**2**	**+36.62%**	**<0.05**	**+31.08%**	**<0.05**
**Cockapoo**	**72**	**6.3**	**3**	**362**	**8.3**	**2**	**183**	**8.8**	**3**	**+39.68%**	**<0.05**	**+6.02%**	**NS**
Border Collie	58	5.1	5	150	3.4	5	87	4.2	4	−17.65%	NS	+23.53%	NS
Crossbreed	29	2.5	8	149	3.5	6	76	3.7	5	+48.0%	NS	+5.71%	NS
Golden Retriever	52	4.6	6	120	2.8	9	75	3.6	6	−21.74%	NS	+28.57%	NS
Cavapoo	20	1.7	12	111	2.6	10	173	2.3	7	+35.29%	NS	−11.54%	NS
**Miniature Smooth-Haired Dachshund**	**64**	**5.6**	**4**	**188**	**4.4**	**4**	**49**	**2.3**	**8**	**−58.93%**	**<0.05**	**−47.73%**	**<0.05**
English Springer Spaniel	24	2.1	10	96	2.2	11	46	2.2	9	+4.76%	NS	0.0%	NS
German Shepherd Dog	27	2.4	9	86	2.0	12	39	1.9	10	−20.83%	NS	−5.0%	NS
**Labradoodle**	**23**	**2.0**	**11**	**127**	**2.9**	**8**	**38**	**1.8**	**11**	**−10.0%**	**NS**	**−37.93%**	**<0.05**
**Border Terrier**	**42**	**3.7**	**7**	**140**	**3.2**	**7**	**29**	**1.4**	**16**	**−62.16%**	**<0.05**	**−56.25%**	**NS**

**Table 2:** Final multinomial model for owner demographic and lifestyle variables associated with purchasing a 2021 puppy or a 2020 “Pandemic Puppy” in the UK (where 2019 is used as the reference value). CI = Confidence interval.

Variable	Category	2020						2021					
		Coeff	SE	OR	(95% CI)		*p*	Coeff	SE	OR	(95% CI)		*p*
					Lower	Upper					Lower	Upper	
Furloughed	Yes	−0.31	0.05	0.74	0.67	0.81	**<0.001**	−0.33	0.06	0.72	0.64	0.81	**<0.001**
	No	Reference											
Keyworker	Yes	0.04	0.05	1.04	0.94	1.14	0.467	0.35	0.05	1.42	1.28	1.58	**<0.001**
	No	Reference											

**Table 3:** Comparison of reasons why UK owners wanted to purchase a dog compared between 2019 puppy (*n* = 1148), 2020 Pandemic Puppy (*n* = 4360) and 2021 puppy (*n* = 2080) owners in the UK. Significant differences at the 5% level identified by (Bonferroni-corrected) post-hoc comparisons are denoted by letters (e.g., a, b, c).

Reason (*n* = 7585)	Acquisition Year			Statistics (Overall)	
	2019 (%) (*n* = 1148)	2020 (%) (*n* = 4360)	2021 (%) (*n* = 2080)	*X* ^2^	*p*-Value
Companionship for myself	64.5	64.7	65.4	0.34	0.845
To encourage myself/my family to walk and exercise	47.6 a	53.6 b	49.3 a	18.76	**<0.001**
To improve my/my family’s mental health	38.1 a	46.9 b	43.0 c	31.14	**<0.001**
Companionship for other adult(s) in my household	34.8 a, b	37.0 a	31.8 b	16.28	**<0.001**
Due to the loss of a previous dog in my household	30.8	28.6	29.0	2.44	0.295
Companionship for my children	19.1 a	24.0 b	19.7 a	22.72	**<0.001**
To keep me/my family busy	22.8	23.7	25.3	2.71	0.257
Companionship for my other dog(s)	25.7 a	19.9 b	21.7 b	17.81	**<0.001**
As a working dog for a specific role (e.g., gundog, security, sniffer/tracking, herding, medical detection, assistance/therapy dog)	10.4 a	7.9 b	7.1 b	11.53	**0.003**
^ For a specific non-working role (e.g., dog sports, showing, etc.)	8.1 a	4.1 b	8.8 a	65.10	**<0.001**
^ Due to the ageing of another dog/other pet in my household	0.9 a	0.7 a	7.7 b	285.55	**<0.001**
^ For breeding	1.1 a, b	0.6 a	2.2 b	33.50	**<0.001**
^ Due to the death of another (non-dog) pet in my household	0.2 a	0.4 a	2.0 b	49.97	**<0.001**
^ Companionship for my other (non-dog) pets	0.0 a	0.0 a	3.3 b	172.25	**<0.001**

N.B. ^ indicates a category that was newly introduced as an MCQ option in the 2021 survey based on free-text responses in 2019–2020 cohorts; thus, due to the difference in data collection method, differences are highlighted in the table for future comparison, but not interpreted in the text.

**Table 4:** Characteristics sought by owners when selecting a particular breed/crossbreed puppy to purchase, with comparisons between 2019 puppy (*n* = 1123), 2020 Pandemic Puppy (*n* = 4281) and 2021 puppy (*n* = 2080) owners in the UK. Significant differences at the 5% level identified by (Bonferroni-corrected) post-hoc comparisons are denoted by letters (e.g., a, b).

Characteristic (*n* = 5404)	Acquisition Year			Statistics	
	2019 (%) (*n* = 1123)	2020 (%) (*n* = 4281)	2021 (%) (*n* = 2080)	*X* ^2^	*p*-Value
Good companion	67.9 a, b	70.8 a	63.7 b	31.61	**<0.001**
Size suited to my lifestyle	59.1 a	64.9 b	57.9 a	33.59	**<0.001**
Generally healthy breed/crossbreed	45.3 a	48.4 a	38.4 b	54.42	**<0.001**
Good with children	38.7 a	47.9 b	42.8 a	36.04	**<0.001**
Easy to train	34.7 a	43.3 b	36.9 a	40.58	**<0.001**
Appearance/looks	39.3 a	39.7 a	34.6 b	15.27	**<0.001**
I’ve owned this breed or crossbreed before	40.1 a	33.0 b	35.0 b	20.66	**<0.001**
Exercise encouragement	29.9 a, b	33.0 a	27.1 b	22.52	**<0.001**
Friends or family currently own this breed/crossbreed	19.8 a	24.0 b	21.4 a, b	11.78	**0.003**
I grew up with or had childhood experiences with this breed/crossbreed	18.5	18.8	16.4	5.21	0.074
Hypoallergenic	15.2 a	18.0 b	15.0 a	11.20	**0.004**
Low grooming needs	14.2	16.4	14.4	5.81	0.055
Long life expectancy	15.9 a	14.2 a, b	12.3 b	7.82	**0.020**
Working ability of the breed/crossbreed	17.5 a	13.2 b	13.9 b	13.84	**<0.001**
Affordable purchase cost of puppies	8.4	9.3	9.5	1.27	0.530
Affordable cost of upkeep	7.7	8.0	6.9	2.07	0.356
Low exercise requirements	5.0	5.5	5.0	1.08	0.582
Popularity of the breed/crossbreed	4.9	4.4	3.5	3.81	0.149
^ Other perceived temperament/personality traits	3.8 a	3.2 a	44.7 b	1995.98	**<0.001**
^ None of these options—I did not have any specific characteristics I was looking for	3.2 a	1.6 b	2.3 a, b	13.11	**<0.001**
^ I’ve always wanted to own this breed/crossbreed	0.5 a	0.5 a	24.8 b	1315.94	**<0.001**
^ Low or non-shedding	0.6 a	0.4 a, b	0.2 b	5.82	0.054
^ Specific genetic characteristics of the breed/crossbreed	0.5 a	0.4 a	16.4 b	827.93	**<0.001**
^ Celebrity /Influencer endorsement/ ownership	0.4	0.1	0.1	5.12	0.077
^ None of these options—someone else in the household selected the breed/crossbreed of our puppy	0.3 a	0.1 a	1.2 b	40.48	**<0.001**

N.B. ^ indicates a category that was newly introduced as an MCQ option in the 2021 survey based on free-text responses in 2019–2020 cohorts; thus, due to the difference in data collection method, differences are highlighted in the table for future comparison, but not interpreted in the text.

**Table 5:** Characteristics of breeders that prospective owners sought out, with comparisons between 2019 puppy (*n* = 1101), 2020 Pandemic Puppy (*n* = 4178) and 2021 puppy (*n* = 2080) owners in the UK. Significant differences at the 5% level identified by (Bonferroni-corrected) post-hoc comparisons are denoted by letters (e.g., a, b, c).

Breeder Characteristic (*n* = 5279)	Acquisition Year			Statistics	
	2019 (%) (*n* = 1101)	2020 (%) (*n* = 4178)	2021 (%) (*n* = 2080)	*X* ^2^	*p*-Value
They would allow me to see the puppies’ mother (dam)	90.5 a	88.9 a	86.1 b	15.40	**<0.001**
Someone I felt cared for their dogs	81.7 a, b	84.6 a	78.8 b	31.28	**<0.001**
Someone I felt was trustworthy	79.2 a	78.9 a	69.1 b	72.83	**<0.001**
Good communication with me	74.2 a	78.8 b	72.9 a	29.76	**<0.001**
They performed health tests for the breed/crossbreed I wanted	66.9 a	62.1 b	58.0 c	23.76	**<0.001**
They would allow me to see the puppies’ father (sire)	44.2 a	42.1 a	38.2 b	12.13	**0.002**
Availability of the breed I wanted	39.2 a	41.8 a	32.2 b	50.04	**<0.001**
Lived within the distance I was willing to travel	37.5	40.5	37.6	6.73	**0.035**
Availability of puppies at the time I wanted	38.3 a	40.1 a	33.7 b	22.44	**<0.001**
Reasonably priced puppies	35.5	36.3	33.2	5.60	0.061
Bred the colour of the breed/crossbreed I wanted to purchase	21.0 a, b	21.8 a	18.8 b	7.59	**0.021**
A member of the Kennel Club Assured Breeders Scheme	25.2 a	20.2 b	18.9 b	18.24	**<0.001**
The dogs they bred from had been awarded prizes at dog shows	11.3 a	8.2 b	7.2 b	15.65	**<0.001**
^ Someone I already knew	1.1 a	1.1 a	16.7 b	696.49	**<0.001**
^ Registered with the local Council	0.5 a	0.6 a	16.1 b	744.51	**<0.001**
^ They bred dogs with specific working and/or sporting characteristics	1.0 a	0.6 a	14.6 b	557.34	**<0.001**
^ They were a member of a specific breed/crossbreed club or association	0.2 a	0.3 a	11.5 b	567.82	**<0.001**
^ They registered their puppies with an international canine registration body (e.g., FCI)	0.0 a	0.1 a	3.1 b	149.75	**<0.001**

N.B. ^ indicates a category that was newly introduced as an MCQ option in the 2021 survey based on free-text responses in 2019–2020 cohorts; thus, due to the difference in data collection method, differences are highlighted in the table for future comparison, but not interpreted in the text.

**Table 6:** Final multinomial model for pre-purchase motivations associated with purchasing a 2021 puppy or a 2020 ‘Pandemic Puppy’ in the UK (where 2019 is used as the reference value). CI = Confidence interval; Cat = category; Coeff = coefficient; KC ABS = Kennel Club Assured Breeders Scheme; Good comms = good communication.

Question	Variable	Cat	2020						2021					
			Coeff	SE	OR	(95% CI)		*p*	Coeff	SE	OR	(95% CI)		*p*
						Lower	Upper					Lower	Upper	
Motivation for dog	Mental health	Yes	0.27	0.70	1.32	1.15	1.52	**0.001**	0.21	0.79	1.23	1.05	1.43	**0.009**
		No	Reference											
Breed characteristics desired	Good with children	Yes	0.31	0.07	1.36	1.19	1.57	**<0.001**	0.22	0.08	1.25	1.07	1.46	**0.005**
		No	Reference											
	Healthy breed	Yes	0.01	0.07	1.01	0.88	1.16	0.908	−0.29	0.08	0.75	0.64	0.88	**<0.001**
		No	Reference											
	Easy to train	Yes	0.27	0.07	1.32	1.14	1.52	**<0.001**	0.15	0.08	1.16	0.99	1.37	0.063
		No	Reference											
Desirable characteristics of breeder	Good comms	Yes	0.31	0.82	1.36	1.16	1.60	**<0.001**	0.10	0.09	1.11	0.93	1.32	0.264
		No	Reference											
	Did health tests	Yes	−0.23	0.07	0.80	0.69	0.92	**0.002**	−0.30	0.08	0.74	0.63	0.87	**<0.001**
		No	Reference											
	Allowed to see Mum	Yes	−0.28	0.12	0.76	0.60	0.96	**0.021**	−0.30	0.13	0.74	0.58	0.96	**0.022**
		No	Reference											
	Allowed to see Dad	Yes	−0.07	0.07	0.94	0.82	1.07	0.331	−0.18	0.08	0.84	0.72	0.98	**0.023**
		No	Reference											
	KC ABS member	Yes	−0.24	0.08	0.79	0.68	0.93	**0.004**	−0.28	0.09	0.76	0.63	0.91	**0.003**
		No	Reference											

**Table 7:** Sources of information used when researching dog ownership and/or which breed/crossbreed to purchase prior to purchasing a puppy, with comparisons between 2019 puppy (*n* = 591), 2020 Pandemic Puppy (*n* = 2771) and 2021 puppy owners (*n* = 1036) in the UK. Significant differences at the 5% level identified by (Bonferroni-corrected) post-hoc comparisons are denoted by letters (e.g., a, b, c).

Information Source (*n* = 3362)	Acquisition Year			Statistics	
	2019 (%) (*n* = 591)	2020 (%) (*n* = 2771)	2021 (%) (*n* = 1036)	*X* ^2^	*p*-Value
Talking to friends or family who own or had owned a dog	54.8 a	65.3 b	59.9 a	27.36	**<0.001**
A breed/crossbreed-specific online resource (e.g., website/forum)	54.8 a	60.1 b	48.1 a	44.90	**<0.001**
The Kennel Club website	50.6 a, b	54.2 a	46.4 b	18.29	**<0.001**
Talking to a dog breeder	53.6 a	49.7 a, b	46.5 b	8.25	**0.016**
Social media sites, e.g., Facebook, Instagram	43.5	47.2	44.5	3.73	0.155
An animal charity website, e.g., Dogs Trust, RSPCA, PDSA, etc.	28.8 a	45.0 b	37.2 c	61.08	**<0.001**
Book(s)	30.1	35.0	34.0	5.03	0.081
My veterinary professional (e.g., veterinary surgeon, veterinary nurse)	14.0	11.3	12.0	3.65	0.162
Dog-specific magazine(s)	6.4	5.4	4.2	3.81	0.149
^ Other digital sources (e.g., articles on the internet, TV shows)	3.7 a	3.2 a	54.7 b	1608.76	**0.001**
^ Talking to a non-veterinary animal professional (e.g., dog trainer, behaviourist)	1.0 a	1.0 a	14.6 b	360.67	**<0.001**
^ Talking to current dog owners that I met (that were not friends or family)	2.4 a	1.0 a	36.4 b	1142.68	**<0.001**
I can’t remember	0.8	0.3	0.2	4.76	0.092
^ From professional experience with dogs in my workplace	0.7 a	0.3 a	16.1 b	502.74	**<0.001**
^ Seeking practical experience of caring for dogs (e.g., dog sitting, fostering)	0.2 a	0.1 a	8.4 b	264.80	**<0.001**

N.B. ^ indicates a category that was newly introduced as an MCQ option in the 2021 survey based on free-text responses in 2019–2020 cohorts; thus, due to the difference in data collection method, differences are highlighted in the table for future comparison, but not interpreted in the text.

**Table 8:** Places owners found their puppy with comparisons between 2019 puppy (*n* = 1100), 2020 Pandemic Puppy (*n* = 4177) and 2021 puppy (*n* = 2080) in the UK. Significant differences at the 5% level identified by (Bonferroni-corrected) post-hoc comparisons are denoted by letters (e.g., a, b).

Method of Initially Finding Puppy (*n* = 7357)	Acquisition Year			Statistics	
	2019 (%) (*n* = 1100)	2020 (%) (*n* = 4177)	2021 (%) (*n* = 2080)	*X* ^2^	*p*-Value
An animal specific selling website, e.g., Pets4Homes, Champdogs	34.5 a	45.0 b	34.9 a	72.78	**<0.001**
I already knew the breeder (e.g., colleague, friends, family, repeat purchase)	26.8 a	19.8 b	21.9 b	26.45	**<0.001**
Recommendation from a friend	11.5 a	14.3 b	14.3 b	6.11	**0.047**
The Kennel Club website ‘Find A Puppy’ search	16.9 a	11.8 b	10.2 b	29.89	**<0.001**
A general selling website, e.g., FreeAds, Gumtree, Preloved	9.3	9.3	8.4	1.57	0.456
A social media breed/crossbreed specific group	8.5 a, b	8.4 a	6.5 b	7.53	**0.023**
The breeder’s website	8.7	7.6	7.2	2.29	0.318
The breeder’s social media account	6.5	6.0	6.9	1.87	0.392
^ Recommendation from another breeder/stud dog owner	1.2 a	2.0 a	7.0 b	119.65	**<0.001**
^ Recommendation from someone who is not a colleague, friend, family member or animal professional	1.9 a, b	1.6 a	3.2 b	17.14	**<0.001**
Dog specific magazine(s)/newspaper(s)	0.3	0.6	0.4	1.84	0.399
^ An advert seen in another online location	0.6 a	0.3 a	7.8 b	343.34	**<0.001**
^ The breeder contacted me directly following general expression of interest for a puppy	0.1	0.3	0.5	5.17	0.075
Local newspaper advert	0.0	0.2	0.0	6.36	0.042
^ Recommendation from an animal professional (e.g., veterinary surgeon, veterinary nurse, dog trainer)	0.2 a	0.2 a	3.0 b	123.29	**<0.001**
^ Recommendation from a stranger	0.2 a	0.2 a	1.1 b	28.18	**<0.001**
An advert in a local shop	0.1	0.0	0.0	2.11	0.349
^ A physical advert in another location (e.g., vets noticeboard)	0.2	0.0	0.4	17.23	**<0.001**

N.B. ^ indicates a category that was newly introduced as an MCQ option in the 2021 survey based on free-text responses in 2019–2020 cohorts; thus, due to the difference in data collection method, differences are highlighted in the table for future comparison, but not interpreted in the text.

**Table 9:** Puppy viewing practices prior to the date the puppy was brought home (*n* = 5280) with comparison between 2019 owners (*n* = 1100) and Pandemic Puppy owners (*n* = 4180) in the UK. Significant differences at the 5% level identified by (Bonferroni-corrected) post-hoc comparisons are denoted by letters (e.g., a, b, c).

Viewing Practice (*n* = 7360)	Acquisition Year			Statistics	
	2019 (%) (*n* = 1100)	2020 (%) (*n* = 4180)	2021 (%) (*n* = 2080)	*X* ^2^	*p*-Value
Yes—visited the breeder’s property in person	80.6 a	59.6 b	70.2 c	193.23	**<0.001**
Yes—saw photos or a pre-recorded video of my/our puppy	31.2 a	51.6 b	39.6 c	175.39	**<0.001**
Yes—saw my/our puppy on a live video call with their breeder	6.5 a	28.9 b	18.2 c	273.58	**<0.001**
No—I did not ask to see my/our puppy	4.7 a	4.9 a	1.1 b	51.59	**<0.001**
^ No—the purchase was a rapid decision, so my puppy was brought home on the same day they were viewed	5.1 a	2.7 b	6.8 a	58.81	**<0.001**
^ No—I was unable to visit due to the breeder being too far away to travel to	1.7 a	2.0 a	6.3 b	88.54	**<0.001**
No—I wanted to see my/our puppy, but the breeder refused	0.1 a	1.1 b	0.3 a	17.26	**<0.001**
^ No—but a friend/relative visited the breeder’s property on my behalf	0.1 a	0.2 a	0.8 b	13.45	**0.001**
^ Yes—I saw my puppy in person but not at the breeder’s property	0.0 a	0.1 a	1.4 b	55.00	**<0.001**
^ No—I only spoke to the breeder by telephone	0.1 a	0.1 a	2.9 b	136.72	**<0.001**
^ No—I asked to visit but personal circumstances meant I was unable to visit myself or get someone else to visit on my behalf	0.1 a	0.0 a	1.2 b	50.12	**<0.001**

N.B. ^ indicates a category that was newly introduced as an MCQ option in the 2021 survey based on free-text responses in 2019–2020 cohorts; thus, due to the difference in data collection method, differences are highlighted in the table for future comparison, but not interpreted in the text.

**Table 10:** Acquisition levels of first-choice breeds by owners and reasons for not purchasing their first-choice breed with comparison between 2019 puppy owners (*n* = 1118), Pandemic Puppy owners (*n* = 4256) and 2021 owners (*n* = 2080) in the UK. Significant differences at the 5% level identified by (Bonferroni-corrected) post-hoc comparisons are denoted by letters (e.g., a, b, c).

First Choice Breed? (*n* = 5374)	Year		
	2019 (%) (*n* = 1118)	2020 (%) (*n* = 4256)	2021 (%) (*n* = 2080)
Yes	91.6 a	86.6 b	80.0 c
No, I could not find a seller that had puppies available at the time for my first choice	1.2 a	3.8 b	3.1 a
No, first choice too expensive	0.8	2.8	1.3
^ No, I/we didn’t have a specific choice in mind	3.0 a	2.7 a	5.5 b
No, I could not find a breeder I felt happy buying my puppy from for my first choice	0.4	1.3	1.6
^ No, I/we changed our mind	1.0	0.8	0.8
^ No, I/we wanted a rescue dog but were unable to source one	0.4 a	0.7 a	4.7 b
^ No, but I had several breeds (≤3) I was interested in and got one of them	0.4 a	0.3 a	2.8 b
No, puppies of my first choice were too far away	0.3	0.2	0.3
^ No, our planned purchase of our first choice fell through	0.3 a, b	0.2 a	0.5 b
^ No, but I had several breeds (>3) that I was interested in and got one of those	0.6 a	0.5 a	0.1 b

N.B. ^ indicates a category that was newly introduced as an MCQ option in the 2021 survey based on free-text responses in 2019–2020 cohorts; thus, due to the difference in data collection method, differences are highlighted in the table for future comparison, but not interpreted in the text.

**Table 11:** Final multinomial model for pre-purchase behaviours associated with purchasing a 2021 puppy or 2020 ‘Pandemic Puppy’ (where 2019 is used as the reference value). CI = Confidence interval. Cat = Category; Coeff = Coefficient.

Question	Variable	Cat	2020						2021					
			Coeff	SE	OR	(95% CI)		*p*	Coeff	SE	OR	(95% CI)		*p*
						Lower	Upper					Lower	Upper	
Pre-purchase information	Charity website	Yes	0.65	0.11	1.91	1.54	2.39	**<0.001**	0.40	0.12	1.50	1.17	1.91	**0.001**
		No	Reference											
	Family/friends	Yes	0.32	0.11	1.37	1.11	1.69	**0.003**	0.12	0.12	1.13	0.90	1.43	0.296
		No	Reference											
Find breeder	Kennel Club Website	Yes	−0.62	0.13	0.54	0.42	0.70	**<0.001**	−0.71	0.15	0.49	0.37	0.66	**<0.001**
		No	Reference											
Viewing prior to purchase	Video recordings and photos	Yes	0.36	0.11	1.44	1.15	1.82	**0.002**	0.35	0.13	1.42	1.11	1.83	**0.005**
		No	Reference											
	Live video calls	Yes	1.21	0.21	3.35	2.23	5.05	**<0.001**	0.68	0.26	1.98	1.27	3.10	**0.003**
		No	Reference											
	Visited in person	Yes	−1.45	0.37	0.23	0.11	0.49	**<0.001**	−1.00	0.40	0.37	0.17	0.80	**0.011**
		No	Reference											
Number of (in person) visits to breeder			−0.12	0.03	0.89	0.83	0.95	**<0.001**	0.02	0.03	1.02	0.97	1.08	0.450

**Table 12:** Locations owners received their puppy with comparison between 2019 puppy owners (*n* = 1100), Pandemic Puppy owners (*n* = 4180) and 2021 puppy owners (*n* = 2080) in the UK. Significant differences at the 5% level identified by (Bonferroni-corrected) post-hoc comparisons are denoted by letters (e.g., a, b, c).

Location (*n* = 5280)	Acquisition Year			Statistics	
	2019 (%) (*n* = 1100)	2020 (%) (*n* = 4180)	2021 (%) (*n* = 1860)	*X* ^2^	*p*-Value
The breeder’s property—from inside their home	84.7 a	51.0 b	67.2 c	464.07	**<0.001**
The breeder’s property—from outside their home, e.g., doorstep, garden	5.5 a	29.8 b	13.7 c	399.36	**<0.001**
The breeder’s property—an outdoor kennels, barn or outbuilding	13.2	14.8	13.4	3.49	0.175
The breeder delivered my puppy to my property	1.0 a	5.2 b	2.1 a	60.73	**<0.001**
A service station	0.0 b	1.4 a	0.1 b	38.24	**<0.001**
A car park	0.3 a	1.1 b	0.1 a	21.31	**<0.001**
^ A courier/pet transporter delivered my puppy to my property	0.5	0.9	0.3	6.45	0.040
^ Breeder/courier met us half way (location unspecified)	0.1 a	0.5 a, b	0.9 b	9.47	**0.009**
^ The breeder’s workplace or non-residential property (e.g., holiday home)	0.3	0.4	0.4	0.41	0.817
^ A veterinary practice	0.1	0.2	0.1	1.33	0.514
^ Other transport location (e.g., train station or ferry port)	0.0	0.2	0.2	2.20	0.333
^ Other public location (e.g., park, shop, hotel)	0.0	0.2	0.4	4.44	0.109
An airport	0.1	0.1	0.2	0.31	0.856
A lay-by	0.0	0.1	0.1	0.80	0.671
^ A friend collected and delivered my puppy for me	0.1	0.0	0.6	34.30	**<0.001**

N.B. ^ indicates a category that was newly introduced as an MCQ option in the 2021 survey based on free-text responses in 2019–2020 cohorts; thus, due to the difference in data collection method, differences are highlighted in the table for future comparison, but not interpreted in the text.

**Table 13:** Owners’ requests for information related to health testing of their puppies’ parents with comparison between 2019 puppy owners (*n* = 908), Pandemic Puppy owners (*n* = 3509) and 2021 dog owners (*n* = 1634) in the UK.

Test Type	Request and Provision of Information	Acquisition Year			Statistics	
		2019 (%) (*n* = 908)	2020 (%) (*n* = 3509)	2021 (%) (*n* = 1634)	*X* ^2^	*p*-Value
Results of DNA (genetic) tests (*n* = 6051)	Yes, and they provided me with it	48.5 a	40.7 b	43.8 a, b	26.16	<0.001
	Yes, but they could not provide me with it	2.6 a, b	4.3 a	2.7 b		
	No, I did not ask about this	41.0 a	46.7 b	45.0 a, b		
	No, I do not believe there are any tests available for my puppy’s breed/crossbreed	8.0	8.2	8.4		
Results of veterinary screening tests (e.g., hips, elbows, knees, eyes, respiratory testing) (*n* = 6051)	Yes, and they provided me with it	58.1 a	46.4 b	51.3 c	45.25	<0.001
	Yes, but they could not provide me with it	3.7	4.8	4.2		
	No, I did not ask about this	30.8 a	41.2 b	37.4 c		
	No, I do not believe there are any tests available for my puppy’s breed/crossbreed	7.4	7.6	7.0		

**Table 14:** Other dogs that were seen at the seller’s premises on the day of purchase of the puppy with comparison between 2019 puppy owners (*n* = 1073), Pandemic Puppy owners (*n* = 4095) and 2021 puppy owners (*n* = 1827) in the UK. Significant differences at the 5% level identified by (Bonferroni-corrected) post-hoc comparisons are denoted by letters (e.g., a, b, c).

Other Dogs and Their Relationship to the Purchased Puppy (*n* = 6995)	Acquisition Year			Statistics	
	2019 (%) (*n* = 1073)	2020 (%) (*n* = 4095)	2021 (%) (*n* = 1827)	*X* ^2^	*p*-Value
Their mother (dam)	85.7 a	75.1 b	84.9 a	106.75	**<0.001**
Their littermates	84.9 a	72.1 b	81.6 a	113.30	**<0.001**
Another dog(s) they were not related to (e.g., another breed)	33.2 a	25.7 b	26.4 b	24.52	**<0.001**
Their father (sire)	26.9 a	23.5 a, b	22.8 b	6.87	**0.032**
I only saw my/our puppy	4.9 a	12.7 b	6.3 a	93.97	**<0.001**
^ Adult dog(s) they were related to (e.g., aunts, grandparents, older siblings)	8.6 a	5.5 b	25.6 c	519.71	**<0.001**
Other puppies (unsure if they were littermates)	4.3 a	2.8 b	0.3 c	52.82	**<0.001**
I’m not sure, I wasn’t the person who collected my/our puppy	0.2 a	0.9 b	0.7 a, b	6.16	**0.046**
^ Other puppies my puppy was related to (but not littermates)	0.4 a	0.3 a	3.1 b	106.85	**<0.001**
I don’t remember	0.2	0.2	0.1	0.55	0.760
^ Adult dog(s) the breeder claimed were my puppy’s parent(s), but I’m not sure	0.3	0.1	0.1	1.32	0.516
^ Other puppies they were not related to (e.g., another breed)	0.0 a	0.0 a	6.2 b	324.68	**<0.001**

N.B. ^ indicates a category that was newly introduced as an MCQ option in the 2021 survey based on free-text responses in 2019–2020 cohorts; thus, due to the difference in data collection method, differences are highlighted in the table for future comparison, but not interpreted in the text.

**Table 15:** Age in weeks when taken home of puppies sold with a Pet Passport in 2019 (*n* = 36), 2020 (*n* = 243) and 2021 (*n* = 169) in the UK. Significant differences at the 5% level identified by (Bonferroni-corrected) post-hoc comparisons are denoted by letters (e.g., a, b).

Age at Acquisition of Puppies Sold with a Pet Passport (*n* = 448)	Acquisition Year		
	2019 (%) (*n* = 36)	2020 (%) (*n* = 243)	2021 (%) (*n* = 169)
13 to 16 weeks old	27.8 a	12.6 a, b	10.7 b
11 to 12 weeks old	2.8 a, b	5.7 a	13.6 b
9 to 10 weeks old	33.3	25.2	25.4
7 to 8 weeks old	36.1	56.5	49.7
Under 6 weeks old	0.0	0.0	0.6

**Table 16:** Reasons owners chose not to or were unable to use The Puppy Contract during their puppy purchase with comparison between 2019 puppy owners (*n* = 151), Pandemic Puppy owners (*n* = 538) and 2021 puppy owners (*n* = 284) in the UK. Significant differences at the 5% level identified by (Bonferroni-corrected) post-hoc comparisons are denoted by letters (e.g., a, b).

Reason for Not Using the Puppy Contract (*n* = 973)	Acquisition Year			Statistics	
	2019 (%) (*n* = 151)	2020 (%) (*n* = 538)	2021 (%) (*n* = 284)	*X* ^2^	*p* Value
I didn’t feel it was needed for the sale of my puppy as I was confident in my own purchasing decision	21.9 a	28.2 a, b	36.3 b	10.79	**0.005**
The breeder did not offer to use The Puppy Contract	20.5	22.4	23.6	0.53	0.767
I used a written contract with the breeder, but not The Puppy Contract	15.9 a, b	12.4 a	19.7 b	8.06	**0.018**
I didn’t feel it was needed, as the breeder is a friend, family member or someone I’ve bought a puppy from before	13.9 a, b	10.2 a	19.7 b	14.71	**<0.001**
I didn’t know enough or feel confident enough about The Puppy Contract to use it	7.2 a, b	6.3 a	11.6 b	7.41	**0.025**
I forgot to use it when purchasing my puppy	2.6 a	6.1 a, b	9.2 b	7.19	**0.027**
I didn’t think it was relevant and/or possible for the sale of my puppy	7.3	5.6	5.6	0.69	0.707
The breeder didn’t agree to use The Puppy Contract when asked	0.7	3.9	1.4	7.11	0.029
I used a verbal contract with the breeder, but not The Puppy Contract:	2.6 a, b	2.2 a	7.0 b	12.82	**0.002**
I felt uncomfortable proposing its use and/or feared repercussions of suggesting it	0.7	2.0	4.2	6.15	0.046
I have negative views on the value of The Puppy Contract	2.0	0.9	0.0	4.96	0.084

**Table 17:** Final multinomial model for purchasing behaviours associated with purchasing a 2021 puppy compared with a 2020 ‘Pandemic Puppy’ or 2019 puppy in the UK (where 2019 is used as the reference value). CI = Confidence interval.

Question.	Variable	Cat	2020						2021					
			Coeff	SE	OR	(95% CI)		*p*	Coeff	SE	OR	(95% CI)		*p*
						Lower	Upper					Lower	Upper	
Collect puppy	Inside of breeders	Yes	−1.34	0.14	0.26	0.20	0.34	**<0.001**	−0.99	0.15	0.37	0.28	0.50	**<0.001**
		No	Reference											
	Outside of breeders	Yes	1.34	0.21	3.83	2.54	5.78	**<0.001**	0.60	0.23	1.82	1.16	2.84	**0.009**
		No	Reference											
Health tests	Yes, and they provided me with it		−1.00	0.11	0.37	0.30	0.46	**<0.001**	−0.77	0.12	0.46	0.37	0.59	**<0.001**
	Yes, but they couldn’t provide it		−0.58	0.25	0.56	0.34	0.92	**0.005**	−0.53	0.27	0.59	0.35	1.01	0.053
	No, I do not believe there are any tests available		−0.02	0.19	0.98	0.68	1.45	0.919	−0.05	0.21	0.95	00.63	1.44	0.815
	No, I did not ask about this		Reference											
Deposit	No—I was not asked to		−0.23	0.17	0.80	0.57	1.11	0.173	0.12	0.19	1.13	0.78	1.63	0.514
	Yes—after I saw my puppy		−0.32	0.16	0.72	0.53	0.99	**0.045**	−0.06	0.18	0.94	0.66	1.33	0.728
	Yes—before I saw my puppy		Reference											
Cost of puppy	Under £500		−4.61	0.36	0.01	0.01	0.02	**<0.001**	−4.74	0.37	0.01	0.01	0.02	**<0.001**
	£500–999		−4.10	0.31	0.02	0.01	0.03	**<0.001**	−4.54	0.32	0.01	0.01	0.02	**<0.001**
	£1000–1499		−3.30	0.31	0.04	0.02	0.07	**<0.001**	−3.66	0.32	0.03	0.01	0.04	**<0.001**
	£1500–1999		−2.12	0.33	0.12	0.06	0.23	**<0.001**	−1.99	0.33	0.14	0.07	0.26	**<0.001**
	>£2000		Reference											
Who was seen with puppy on collection	Litter-mates	Yes	−0.51	0.13	0.60	0.46	0.79	**<0.001**	0.01	0.15	1.00	0.75	1.34	0.989
		No	Reference											
	Father	Yes	0.27	0.11	1.31	1.05	1.62	**<0.001**	0.10	0.12	1.10	0.87	1.40	0.426
		No	Reference											

**Table 18:** Reasons why the COVID-19 Pandemic influenced decisions to purchase a puppy for 2020 Pandemic Puppy owners (*n* = 1979) and 2021 puppy owners (*n* = 383) in the UK.

Reason (*n* = 1979)	2020 (*n* = 1979)		2021 (*n* = 383)		Statistics	
	%	*n*	%	*n*	*X* ^2^	*p* Value
I/we had more time to care for a dog	86.7	1378	76.8	294	23.73	**<0.001**
I/we wanted something happy to focus on	30.0	477	25.1	96	3.67	0.055
I/we wanted a reason to go outside to exercise more	29.6	470	28.7	110	0.11	0.741
I/we wanted more company due to being at home more	24.5	390	26.4	101	0.55	0.458
I/we had extra money to spend that I/we would have usually spent on other things	17.7	282	14.6	56	2.12	0.145
I/we wanted more company as family and/or friends were unable to visit me/us	9.1	144	6.0	23	3.72	0.054
I/we were bored due to the restrictions imposed by lockdown	4.3	69	1.8	7	5.27	**0.022**
My child/children were at home, and I/we wanted something to keep them busy	4.1	65	2.6	10	1.85	0.174
^ I/we experienced mental health challenges due to the Pandemic, that I/we wanted a puppy to help us with	0.9	15	21.9	84	285.10	**<0.001**
^ I/we wanted a dog from another source (e.g., rescue) but were unable to due to Pandemic, so bought a puppy instead	0.8	13	4.7	18	30.05	**<0.001**
^ My/our existing dog experienced mental health challenges due to the Pandemic, that I/we wanted a puppy to help them with	0.1	2	2.3	9	27.52	**<0.001**

N.B. ^ indicates a category that was newly introduced as an MCQ option in the 2021 survey based on free-text responses in the 2020 cohort; thus, due to the difference in data collection method, differences are highlighted in the table for future comparison, but not interpreted in the text.

## Data Availability

Anonymous data, which cannot be traced to any individual, that are presented in this study are available upon request for future research from the corresponding author.
